# Common and rare variants associating with serum levels of creatine kinase and lactate dehydrogenase

**DOI:** 10.1038/ncomms10572

**Published:** 2016-02-03

**Authors:** Ragnar P. Kristjansson, Asmundur Oddsson, Hannes Helgason, Gardar Sveinbjornsson, Gudny A. Arnadottir, Brynjar O. Jensson, Aslaug Jonasdottir, Adalbjorg Jonasdottir, G. Bragi Walters, Gerald Sulem, Arna Oskarsdottir, Stefania Benonisdottir, Olafur B. Davidsson, Gisli Masson, Olafur Th Magnusson, Hilma Holm, Olof Sigurdardottir, Ingileif Jonsdottir, Gudmundur I. Eyjolfsson, Isleifur Olafsson, Daniel F. Gudbjartsson, Unnur Thorsteinsdottir, Patrick Sulem, Kari Stefansson

**Affiliations:** 1 deCODE genetics/Amgen, Inc., 101 Reykjavik, Iceland; 2 School of Engineering and Natural Sciences, University of Iceland, 101 Reykjavik, Iceland; 3 Department of Internal Medicine, Landspitali The National University Hospital of Iceland, 101 Reykjavik, Iceland; 4 Department of Clinical Biochemistry, Akureyri Hospital, 600 Akureyri, Iceland; 5 Icelandic Medical Center (Laeknasetrid), Laboratory in Mjodd (RAM), 109 Reykjavik, Iceland; 6 Department of Clinical Biochemistry, Landspitali University Hospital, 101 Reykjavik, Iceland; 7 Faculty of Medicine, University of Iceland, 101 Reykjavik, Iceland

## Abstract

Creatine kinase (CK) and lactate dehydrogenase (LDH) are widely used markers of tissue damage. To search for sequence variants influencing serum levels of CK and LDH, 28.3 million sequence variants identified through whole-genome sequencing of 2,636 Icelanders were imputed into 63,159 and 98,585 people with CK and LDH measurements, respectively. Here we describe 13 variants associating with serum CK and 16 with LDH levels, including four that associate with both. Among those, 15 are non-synonymous variants and 12 have a minor allele frequency below 5%. We report sequence variants in genes encoding the enzymes being measured (*CKM* and *LDHA*), as well as in genes linked to muscular (*ANO5*) and immune/inflammatory function (*CD163/CD163L1, CSF1, CFH, HLA-DQB1, LILRB5, NINJ1* and *STAB1*). A number of the genes are linked to the mononuclear/phagocyte system and clearance of enzymes from the serum. This highlights the variety in the sources of normal diversity in serum levels of enzymes.

## Abstract (web summary)

Creatine kinase (CK) and lactate dehydrogenase (LDH) are biomarkers of tissue damages including myopathy and myocardial infarction. Here, Patrick Sulem and colleagues perform a genome-wide association study to identify common and rare genetic variants that associates with serum CK or LDH levels.

## Graphical abstract

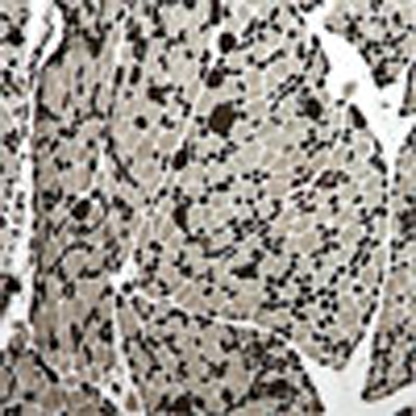

The release of intracellular enzymes into the serum is an indicator of tissue damage and physiological cell turnover. Thus, measurements of intracellular enzymes in serum are widely used to diagnose tissue damage, monitor its course and severity and gauge the effect of therapy.

Creatine kinase (CK) is an enzyme, catalyzing the ATP-dependent phosphorylation of creatine that is important for energy buffering in tissues with variable energy demands, most notably skeletal and cardiac muscle^1^. Elevated serum CK levels can indicate tissue damage, and are observed in a number of pathological conditions, including statin-induced myopathy^2^. Monitoring changes in serum CK levels is therefore important in statin-treated patients who display muscle pain or weakness^3^, and in patients deemed at risk of rhabdomyolysis for various reasons^4^.

Lactate dehydrogenase (LDH) is an enzyme with a ubiquitous expression^5^. It is responsible for catalyzing the anaerobic, nicotinamide adenine dinucleotide phosphate-dependent conversion of pyruvate to lactate, which is important during times of high muscular activity^6^. Serum CK and LDH levels were previously used as biomarkers to diagnose myocardial infarction. Because of low specificity, however, they have been replaced by troponin T and troponin I^7^, measured through high-sensitivity assays^8^.

The heritability of LDH levels has been estimated between 40 and 50% (refs 9, 10) and upwards of 38% for CK (ref. 11). Our own data indicates a 19.33 and 19.36% heritability for CK and LDH, respectively. A recently published genome-wide association study (GWAS) of 3,232,779 imputed variants in 3,412 statin users found two missense variants affecting serum CK levels, one in *CKM* (rs11559024) and one in *LILRB5* (rs12975366) (ref. 12). An association of a variant in *CD163* (rs7136716) with serum CK levels has also been reported^13^. To date, no systematic GWAS has been carried out to search for sequence variants influencing serum LDH levels.

To search for common and rare variants that associate with CK or LDH levels, we tested variants detected in a large sequencing study in Iceland for association with these traits^14^. This unbiased approach has the potential to uncover previously unexpected molecular mechanisms regulating levels of this enzyme in serum. A thorough understanding of sequence variants influencing these biomarkers is important to improve the usefulness of their measurements, and in the assessment of tissue damage^15^.

## Results

### Summary of findings

To search for sequence variants associating with mean serum levels of CK and/or LDH, we analysed 28.3 million variants, initially identified through whole-genome sequencing of 2,636 Icelanders and subsequently imputed into chip-typed individuals through long-range haplotype phasing^16^. Genotype probabilities were calculated for first and second-degree relatives of chip-typed individuals^17^. We tested for association between sequence variants and serum CK in 63,159 individuals (35,623 chip-typed and 27,536 with chip-typed first or second-degree relatives), and serum LDH in 98,585 individuals (52,581 chip-typed and 46,004 with chip-typed first- or second-degree relatives).

We used methodology outlined by Sveinbjornsson *et al*.^18^ to determine weighted genome-wide significance thresholds for different variant classes based on the total number of variants tested per class: loss-of-function (*N*=6,476; *P*=2.6 × 10^−6^); missense (*N*=100,502; *P*=1.7 × 10^−7^); and other (*N*=23,854,999; *P*=7.0 × 10^−10^).

**Figure 1:**
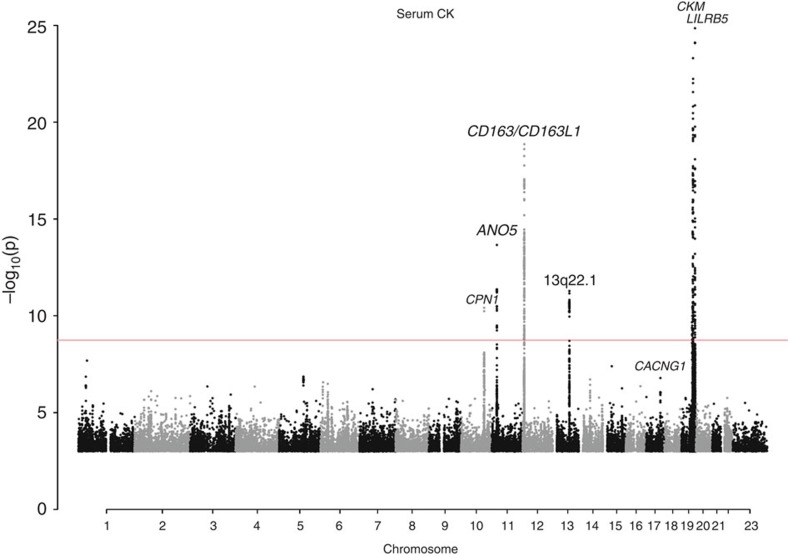
Manhattan plot showing the seven loci harbouring genome-wide significant signals influencing serum CK levels in the Icelandic population. Variants are plotted by chromosomal position (*x* axis) and −log_10_ *P* values (*y* axis). *P* values above 1 × 10^−25^ are represented. Two loci (*CKM* and *LILRB5*) harbour variants with *P* values below this cutoff (rs11559024: *P*=1.8 × 10^−115^; rs12975366: *P*=6.5 × 10^−44^ and rs393600: *P*=1.4 × 10^−33^). The red line indicates the threshold for genome-wide significance, determined by the number of tests performed (*P*=0.05/28.3 million=1.8 × 10^−9^).

**Figure 2:**
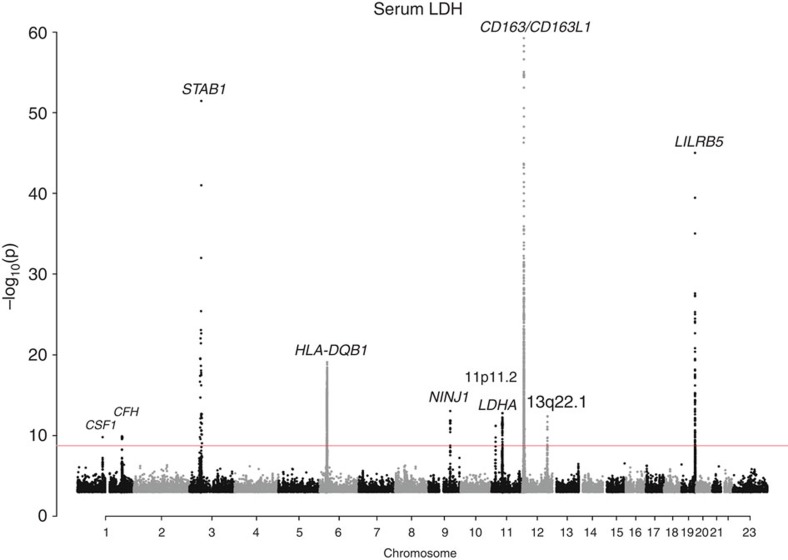
Manhattan plot showing the 10 loci harbouring genome-wide significant signals influencing serum LDH levels in the Icelandic population. Variants are plotted by chromosomal position (*x* axis) and −log_10_ *P* values (*y* axis). *P* values above 1 × 10^−60^ are represented. One locus (*CD163L1*) harbours a variant with *P* value below this cutoff (rs4072797: *P*=9.9 × 10^−89^). The red line indicates the threshold for genome-wide significance, determined by the number of tests performed (*P*=0.05/28.3 million=1.8 × 10^−9^).

We found a total of 13 variants associated with CK, and 16 associated with LDH. Of those, four were associated with both enzymes (Table 1, Figs 1 and 2). In total, all of the reported sequence variants explain 1.9% of CK variance, and 1.8% of LDH variance. Our results include replication of variants in *CKM, LILRB5* and *CD163* reported in GWASs of serum CK levels^12^,^13^. None of the variants showed more significant associations when alternate inheritance models were tested (Supplementary Tables 2 and 3), and no additional variants were detected using these models.

Eleven of the variants are common (minor allele frequency (MAF)>5%), eight are of low frequency (MAF=0.5-5%) and five are rare (MAF<0.5%). Five loci contain several independent signals, associating with either one or both of the enzymes measured (Fig. 3). For loci with multiple signals, we present *P* values and effects before and after adjusting for other significant markers at that locus (Table 1). All variants correlated (*r*^2^>0.6) with the variants we report are shown in the Supplementary Data 1 and Supplementary Figs 5–33.

Most of the suspected genes are implicated in immune/inflammatory response, enzyme clearance or muscular function, but two encode subunits of the enzymes measured (*cis* signals) and one encodes a protein that affects rates of CK clearance from plasma.

### *Cis* signals

We observed variants in *CKM* and *LDHA*, genes that encode subunits of CK and LDH, respectively, with minor alleles that associate with a lower level of their corresponding serum enzyme levels. Enzymatic levels were measured by assessing quantity through enzymatic activity. Our results, therefore, indicate that these sequence variants act to either decrease the amount of the enzyme produced, or their catalytic ability.

#### CKM

Through a stepwise conditional analysis at 19q13.3, we found that four independent missense variants in *CKM* associate with serum CK levels. With one of these, rs11559024 [C] (MAF=2.15%, Glu83Gly), we replicated a reported association^12^. The associations of the remaining three low-frequency and rare missense variants are novel. Minor alleles of all four *CKM* variants have a large lowering effect on CK levels. We never observed more than one of the minor alleles on the same chromosome (all *r*^2^≤2.2 × 10^−4^; Supplementary Table 4).

*CKM* encodes CK-M, one of two subunits of the CK dimer. Three isoforms of the enzyme exist, consisting of different combinations of CK-M and/or CK-B. Each isozyme has a unique expression profile; CK-MM is expressed in skeletal muscle, CK-MB in cardiac muscle and CK-BB in smooth muscle and the brain^19^. CK-MM typically accounts for the majority of serum CK (ref. 20).

#### LDHA

The missense variant rs116841148 [T] (MAF=0.652%, Ala147Ser) at 11p15.1 in *LDHA* associates with serum LDH levels (Table 1). *LDHA* encodes the M subunit of the LDH enzyme, expressed in all but one of the five isozymes of LDH (ref. 21).

#### CPN1

At 10q24.2, the low-frequency missense variant rs61751507 [T] (MAF=4.06%, Gly178Asp) in *CPN1* associates with lower CK levels (Table 1). *CPN1* encodes carboxypeptidase N. This protein is expressed in blood, hydrolyzes CK-MM_1_′s C-terminal lysine and converts CK-MM_1_, the enzyme‘s unaltered form as expressed in tissue, into either CK-MM_2_ or CK-MM_3_. This hydrolyzation alters CK-MM's isoelectric point and half-life without affecting properties such as enzymatic activity^22^,^23^. It is, therefore, plausible that if the variant in *CPN1* induces a change in activity of carboxypeptidase N, it would affect CK-MM clearance rather than having a direct effect on enzymatic activity.

### Immune system genes associating with CK and/or LDH levels

Seven loci harbouring variants associating with serum CK and/or LDH levels have genes that are likely to be responsible for the associations that are involved in immune or inflammatory response; *CSF1*, *CD163/CD163L1*, *STAB1*, *CFH*, *LILRB5*, *HLA-DQB1* and *NINJ1*. Three of these genes (*CSF1, CD163* and *STAB1*) have direct links to the clearance of products of cell lysis from the serum through the mononuclear phagocytic system (MPS) (refs 24, 25, 26). The remaining four implicated genes do not have a known link to enzyme clearance, although most are preferentially expressed in cells of the myeloid lineage.

#### CSF1

At 1p13.3, the intronic variant **rs333947** [A] (MAF=14.92%) in *CSF1* associates with lower LDH levels (Table 1). We observed no correlated coding variants (*r*^2^>0.6) that could explain the association (Supplementary Data 1). The position shows a high degree of evolutionary conservation (GERP (ref. 27) =4.83; Supplementary Table 5). *CSF1* encodes human macrophage-specific colony stimulating factor, a cytokine necessary for the differentiation of monocyte lineage cells, including hepatic Kupffer cells (KCs) (ref. 24). In animal models, reducing KC numbers affects serum enzyme levels, including CK, LDH and aspartate transaminase (AST), without evidence of skeletal or liver damage^24^. Rs333947 [A] also associates with lower AST levels (Supplementary Table 6). We also observed a suggestive association with CK levels. The associations we observed are consistent with *CSF1* function and may indicate an increased macrophage activity, promoting faster clearance of serum enzymes.

We assessed all sequence variants discussed in this paper for associations with AST levels (Supplementary Table 6). Seven variants, including rs333947, associate with AST based on the number of tests performed (*P*<0.05/25=2.0 × 10^−3^). The direction of effect was always consistent; each allele either had an increasing or decreasing effect on the levels of all enzymes it associates with.

#### CD163/CD163L1

At 12p13.31, the region containing *CD163* and its paralog *CD163L1*, we observed associations with CK and LDH levels (Table 1). Two variants associated with both CK and LDH levels and four only with LDH levels.

Two common variants associate with higher serum levels of CK and LDH; the intergenic variant rs7305678 [T] (MAF=16.16), and the intronic rs117692263 [C] (MAF=9.31%). Three low-frequency missense variants in the region associate solely with serum LDH levels; rs4072797 [T] (MAF=4.21%, Asp588Asn) and rs145411783 [A] (MAF=0.66%) in *CD163L1*, and rs4883263 [T] (MAF=3.73%, Ile342Val) in *CD163*. Finally, a common intergenic indel (chr12:7282745:0:TA MAF=21.13%) associates with LDH levels.

*CD163* encodes a scavenger receptor expressed on macrophages and monocytes, including KCs, that is responsible for uptake of the haemoglobin–haptoglobin complex from the bloodstream^25^. The *CD163L1* gene, encoding the M160 receptor, is closely related to *CD163*. The paralog is expressed by many of the same cells and has a sequence that is highly similar to that of *CD163* (ref. 28), but does not have affinity for the same ligands as *CD163* (ref. 25).

Association of the intergenic variant rs7305678 with serum CK levels replicates a previously published association between a single marker, rs7136716 at the *CD163* locus and serum CK levels in the Japanese^13^. Rs7136716 correlates with rs7305678 in both the Chinese/Japanese (*r*^2^=0.85^29^) and Icelandic (*r*^2^=0.75) populations, but is independent of other signals we report in the region.

#### STAB1

At 3p21.1, the very rare missense variant rs150956780 [C] (MAF=0.078%, Val1522Leu) at a highly conserved site within *STAB1* (GERP (ref. 27)=4.56) associates with drastically lower serum LDH levels (effect=−1.526SD) (Table 1). An association signal was observed across a large region and rs150956780 is more frequent outside of Iceland (Tuscany (TSI) MAF=1.0%, CEPH Utah (CEU) MAF=0.6% (ref. 30); Supplementary Table 5). This could reflect a recent introduction of the sequence variant into the Icelandic population. *STAB1* encodes stabilin-1, a transmembrane scavenger receptor expressed in a number of tissues including activated macrophages^26^.

#### CFH

At 1q31.3, in a locus containing the *CFH* gene, the synonymous variant rs2274700 [A] (MAF=38.62%) associates with lower LDH levels (Table 1). Rs2274700 is fully correlated (*r*^2^=1.00) with rs1410996 [A] (MAF=38.58%). Rs1410996 is one of a large number of sequence variants within genes of the complement system reported to associate with age-related macular degeneration^31^,^32^. In our data, the [A] allele associates with lower risk of associate with age-related macular degeneration. Mutations in *CFH* have also been shown to cause both atypical haemolytic uraemic syndrome^33^. *CFH* encodes complement factor H (FH), a key regulator of the alternative pathway of the complement system, produced and secreted in abundance by KCs^34^. FH also binds to long pentraxin 3 (PTX3) through two of FH's SCR domains (SCR7 and SCR19-20)^35^. PTX3 influences the regulation of the complement system^36^ and plays a non-redundant role in the orchestration of tissue repair and remodelling^37^. FH's specific functions and its associations suggest that alterations of enzyme levels occur through modulation of complement activation or tissue repair and remodelling^38^.

#### LILRB5

At 19q13.42, a locus containing *LILRB5*, we observed associations of two common and modestly correlated (*r*^2^=0.24) sequence variants with CK and LDH levels. The strongest signal is a missense variant rs12975366 [C] (MAF=41.62%, Asp247Gly, Asp147Gly) that associates with lower levels of both CK and LDH. A second marker, the intronic variant rs393600 [G] (MAF=25.17%) in *LILRB5*, similarly associates with both CK and LDH levels. *LILRB5*, encoding leukocyte immunoglobulin-like receptor subfamily B member 5, belongs to the LILR class of genes expressed on cells of myeloid lineage, and is involved in inhibition of inflammatory responses^39^,^40^. Showing association of the marker rs12975366 with CK at *LILRB5* replicates previous findings^12^.

#### HLA-DQB1

In the human leukocyte antigen (HLA) region at 6p21.3, the best association signal with LDH levels is represented by the missense variant rs17412833 [T] (MAF=13.76%, Phe119Tyr) in *HLA-DQB1*. The alleles of the different HLA genes are strongly correlated to each other and often discussed as long haplotypes^41^. The HLA genes control the adaptive immune response through presentation of antigens to T cells^42^. We tested imputed HLA alleles of six of the classical HLA genes: *HLA-A*; *HLA-B*; *HLA-C*; *HLA-DQA1*; *HLA-DQB1*; and *HLA-DRB1* for association^43^. The HLA molecular type associating most strongly with serum LDH is HLA-DQB1*06:04 (*r*^2^=0.53). We note that the [T] allele of rs17412833 is present in the following imputed HLA molecular types: DQB1*05:01; 05:02; 05:03; 06:04; and 06:09. *HLA-DQB1* forms a part of the dimeric HLA-DQ molecule^44^. HLA-DQB1*06:04 has previously been implicated in myasthenia gravis in the Chinese^45^ as well as cervical dystonia^46^. Conditional analysis shows that the HLA-DQB1*06:04 signal is fully explained by the missense variant rs17412833, but indicates that the association of rs17412833 is not explained by the HLA-DQB1*06:04 signal.

#### NINJ1

At 9q22.31, the common intron variant rs12342201 [A] (MAF=49.45%) in *NINJ1* associates with lower LDH levels (Table 1). *NINJ1* encodes the ninjurin-1 protein, an adhesion molecule reported to be upregulated in myeloid cells during inflammation and important in immune cell migration following neuronal injury^47^. *NINJ1* expression is ubiquitous, and it has been implicated in liver function and hepatocellular senescence^48^.

### Muscle-linked genes associating with CK and/or LDH levels

Variants at two loci are coding variants in genes that are preferentially expressed in muscle and play a role in its function.

#### ANO5

At 11p14.3, we observed three variants in *ANO5* that associate with serum CK levels. The common missense variant rs7481951 [A] (MAF=37.45%, Leu322Phe) associates with lower serum CK levels. The remaining two rare variants, the missense variant rs137854526 (MAF=0.24%, Phe578Ser), and the nonsense variant chr11:22241070:S (MAF=0.27%, Cys601X), associates with higher CK levels (*P*<0.05/15 non-synonymous=3.3 × 10^−3^). Rs137854526 occurs at a highly conserved site (GERP (ref. 27) =5.69). *ANO5* encodes the protein anoctamin-5, a chloride channel in the endoplasmic reticulum membrane^49^, expressed mainly in skeletal and cardiac muscle. Variants in the gene, including these two rare mutations, have been reported to cause limb-girdle muscular dystrophy, which primarily affects skeletal muscle but often presents with cardiac pathologies^50^,^51^. Chr11:22241070:S shows suggestive association with heart failure (effect=1.629, *P*=1.0 × 10^−3^, *N*=11,374 cases).

Two cases of compound heterozygotes for *ANO5* mutations presenting with skeletal muscle dysfunction and cardiac abnormalities have been described in the literature^49^,^52^. We support this with a further two homozygotes for rare *ANO5* mutations presenting with cardiovascular phenotypes. Firstly, an individual homozygous for rs137854526 and, secondly, an individual homozygous for chr11:22241070, both presenting with several cardiovascular phenotypes (Supplementary Table 7). These findings provide further evidence for the effect of *ANO5* variants on dysfunctions of the heart.

We note another coding variant in a gene specifically expressed in muscle has a *P* value just above the significance threshold. The common missense variant in *CACNG1* rs1799938 [A] (MAF=10.64%, Gly196Ser) suggestively associates with CK levels (*P*=3.2 × 10^−7^; effect=0.046). *CACNG1* encodes the γ subunit of a 1,4-dihydropyridine (DHPR) -sensitive calcium channel, preferentially expressed in skeletal muscle^53^.

### Other signals associating with CK and/or LDH Levels

The three remaining association signals are at loci without any obvious causative gene.

We observed a signal at 11p11.2 associating with lower serum LDH levels, represented by a large group of 276 correlated markers (*r*^2^>0.6; Supplementary Data 1). The marker showing the strongest association is the common intergenic variant rs2930191 [A] (MAF=37.22%).

At 12q24.13, the low-frequency variant chr12:110830276:S (MAF=2.09%) associates significantly with lower LDH levels, and suggestively with CK levels.

Finally, at 13q22.1, the common variant rs7318906 [A] (MAF=47.08%) associates with decreased CK levels.

We used GTEx Portal's eQTL Browser to assess whether any of our reported variants affected gene expression. None of the 25 markers we discussed in the current study was directly reported or correlated (*r*^2^>0.2) to any GTEX cis-eqtl (*P*<1 × 10^–5^).

### Signals observed specifically in statin takers

We examined statin usage, stratifying our CK and LDH measurements by statin intake. Prescription data for statins were available for a period of time ranging from 2003 to 2009 and the stratified analysis therefore only included blood measurements from this interval. No additional signals were detected (Supplementary Figs 1–4). Our data showed no indication of the overall results being driven by individuals treated by statins, or any genetic susceptibility to statin side effects, for any of the loci in question (Supplementary Tables 8 and 9). Furthermore, the reported variant rs4363657 in *SLCO1B1* showed no effect on any of the tested phenotypes (Supplementary Table 10).

### Advantages of whole-genome sequencing

For the 25 reported variants, twenty were present in the 1000G data set (Supplementary Table 11). Of these, three could not be imputed, and a further three showed very low correlation (*r*^2^<0.64) between the directly typed and the 1000G imputed phenotypes. Fourteen variants showed high correlation between directly typed and imputed genotypes (*r*^2^⩾0.8) and could therefore plausibly have been discovered using only 1000G imputation.

Five variants were not present in 1000G, but had correlates (*r*^2^>0.6) within the data set (Supplementary Table 12). Of these, four showed good correlation (*r*^2^>0.6) between imputed and correlated genotypes, and could, therefore, have shown association with CK or LDH levels in a study relying purely on a 1000G imputation data set.

We therefore report seven variants that could not have been discovered using a 1000G imputation set.

## Discussion

Elevation of serum enzyme levels can be a result of two separate processes; increased leakage from tissue into the serum, or reduced clearance^54^. Furthermore, observations of altered serum enzyme levels can result from a change in enzymatic activity. We report variants affecting serum enzyme levels through all three mechanisms.

Our discovery of a high number of genes linked to immune response is consistent with the MPS's role in clearing debris and foreign material from the bloodstream, including through receptor-mediated endocytosis of short-lived cellular enzymes^55^,^56^. Although the MPS has been implicated, the specific receptors responsible for uptake of serum enzymes have not yet been identified^24^. Nonspecific receptors, responsible for uptake of several enzymes, have been postulated^24^,^57^. We report a number of variants in genes expressed preferentially in phagocytic cells, notably hepatic KCs, which affect levels of serum enzymes. The protein products encoded by *CD163* and *STAB1*, and *LILRB5* are all scavenger receptors expressed by macrophages^58^,^59^, and could be the unidentified receptors responsible for endocytosis of serum enzymes by cells of the MPS.

Non-synonymous variants in *ANO5* associates with serum CK levels, consistent with the fact that serum CK levels are largely influenced by leakage of CK from damaged myocytes^60^.

The results of this study underscore the diversity in sources of variation of serum enzyme levels, each of which are influenced by sequence variants, and must be kept in mind when interpreting the measurements.

## Methods

### Population

Measurements of serum CK levels were available for a total of 63,159 Icelanders. Of these, 35,623 were genotyped using Illumina chips and imputed using long-range-phased haplotypes. Genotype probabilities were calculated for 27,536 individuals based on genetic information available for first and second-degree relatives. Measurements of serum LDH levels were available for a total of 98,585 individuals, 52,581 of whom were chip-typed using Illumina chips, and 46,004 of whom had genotype probabilities calculated. All individuals had provided consent, and the study was approved by the Data Protection Commission of Iceland and the Icelandic National Bioethics Committee.

### Stratification by statin intake

CK and LDH measurements were stratified by statin intake. CK measurements were available for 8,900 statin takers (CK_on statin_=5,207; CK_off statin_=6,877), and LDH measurements were available for 9,851 statin takers (LDH_on statin_=6,266; LDH_off statin_=6,877), respectively, with an overlap of 7,534 individuals. These cohorts were used to test sequence variants for association with serum enzyme levels during and outside of times of statin use.

### Serum enzyme measurements

Serum enzyme measurements were obtained from three laboratories in Iceland: The Laboratory of the Icelandic National University Hospital; The Icelandic Medical Center Laboratory in Mjodd; and Akureyri Hospital. We used measurements of serum CK (*N*=63,159, geometric mean=211.1), and LDH (*N*=98,585, geometric mean=113.1). Additional measurement characteristics can be found in Supplementary Table 13. Serum levels were adjusted for sex, age and laboratory of origin. When multiple measurements were available for an individual, the mean adjusted value was used.

### Whole-genome sequencing and Illumina single-nucleotide polymorphism chip genotyping

The process used to whole-genome sequence the 2,636 Icelanders, and the subsequent imputation from which the data for this analysis were generated has been extensively described in a recent publication^14^.

### Association analysis

All serum enzyme measurements were corrected for age, sexand laboratory of origin, and were subsequently standardized to have a normal distribution. To test for association between quantitative traits and sequence variants, a generalized form of linear regression was used (see Supplementary Note 1). The quantitative trait was used as the response variable and the expected allele count (gene dosage) as the covariate.

In the regression analysis of CK/LDH, we used genealogy information and assume a variance covariance matrix proportional to the kinship matrix (see Supplementary Note 1). To account for relatedness and stratification within the case and control sample sets, we applied the method of genomic control^61^. The inflation factor *λ*_g_ of the *χ*^2^ statistics was estimated on the basis of a set of about 300,000 common variants distributed across the genome, and *P* values were adjusted by dividing the corresponding *χ*^2^ values by this factor. The genomic control was calculated to be *λ*=1.16 for CK and 1.22 for LDH (Supplementary Figs 34 and 35).

### Significance thresholds

Sequence variants were weighted according to their prior probability of affecting gene function. Thresholds for genome-wide significance were applied, depending on variant class, as described by Sveinbjornsson *et al*.^18^. The type I error rate of 0.05 was allocated equally between three classes of variants; loss-of-function (*N*=6,476), missense (*N*=100,502) and other variants (*N*=23,854,999). This yielded class-specific Bonferroni genome-wide significance thresholds of 2.6 × 10^−6^, 1.7 × 10^−7^ and 7.0 × 10^−10^, respectively.

### Sanger sequencing and reimputation

Two sequence variants were poorly imputed due to a low number of sequenced carriers in the original Icelandic data set. A group of suspected carriers and non-carriers of the missese variants rs145987658 in *CKM* (info=0.63) and rs150956780 in *STAB1* (info=0.58) were Sanger sequenced, and reimputation was subsequently carried out. Imputation information following reimputation increased for both variants.

### Fraction of variance explained

Fractions of CK and LDH variance explained by the reported variants were calculated using the formula 2*f* (1−*f*) *a*^2^, where *f*=MAF and *a*=effect.

## Additional information

**Accession codes:** European Variant Archive: PRJEB8636.

**How to cite this article:** Kristjansson, R. P. *et al*. Common and rare variants associating with serum levels of creatine kinase and lactate dehydrogenase. *Nat. Commun.* 7:10572 doi: 10.1038/ncomms10572 (2016).

## Supplementary Material

Supplementary Information Supplementary Figures 1-35, Supplementary Tables 1-13, Supplementary Note 1 and Supplementary References

Supplementary Data 1 Equivalence classes of reported variants. Defined as sequence variants showing high correlation (r2 > 0.6) with the leading variant.

## Figures and Tables

**Figure 3:**
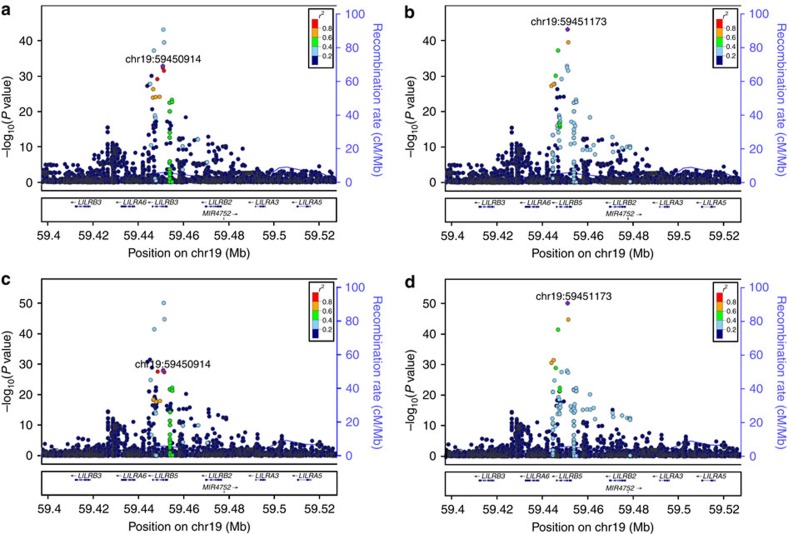
Locus plots depicting variants at the LILRB5 locus associating with serum enzyme levels. Leading variants are labelled and shown in purple, other variants are coloured according to correlation (*r*^2^) with the leading marker (legend at top-right). −log_10_ *P* values are shown along the left *y* axis and correspond to the variants depicted in the plot. The right *y* axis shows calculated recombination rates at the chromosomal location, plotted as a solid blue line. (**a**) Association between the signal represented by rs393600 and serum CK levels. (**b**) Association between the signal represented by rs12975366 and serum CK levels. (**c**) Association between the signal represented by rs393600 and serum LDH levels. (**d**) Association between the signal represented by rs12975366 and serum LDH levels.

**Table 1:** Summary of single marker associations for CK (*N*=63,159) and LDH (*N*=98,585) in Iceland.

**Variant**	**Allele (min/maj)**	**MAF (%)**	Gene*	**CK**				**LDH**			
				**Unadjusted**		**Adjusted**†		**Unadjusted**		**Adjusted**†	
				***P*** **value**	**Effect (Amin)**	***P*** **value**	**Effect (Amin)**	***P*** **value**	**Effect (Amin)**	***P*** **value**	**Effect (Amin)**
rs333947	A/G	14.92	*CSF1*	6.8 × 10^−3^	−0.021	—	—	**2.8** × **10**^−**10**^	−**0.042**	—	—
rs2274700	A/G	38.62	*CFH*	0.58	−0.003	—	—	**4.1** × **10**^−**12**^	−**0.034**	—	—
rs150956780‡	C/G	0.078	*STAB1*	0.60	0.05	—	—	**1.3** × **10**^−**61**^	−**1.526**	—	—
rs17412833‡	T/A	13.76	*HLA-DQB1*	**1.5** × **10**^−**4**^	0.032	—	—	**1.5** × **10**^−**22**^	**0.07**	—	—
rs12342201	A/G	49.45	*NINJ1*	0.70	0.002	—	—	**1.1** × **10**^−**12**^	−**0.034**	—	—
rs61751507‡	T/C	4.06	*CPN1*	**5.1** × **10**^−**11**^	−**0.091**	—	—	0.34	0.011	—	—
rs116841148‡	T/G	0.65	*LDHA*	0.17	0.049	—	—	**2.9** × **10**^−**11**^	−**0.198**	—	—
rs7481951‡	A/T	37.45	*ANO5*	**6.4** × **10**^−**16**^	−**0.047**	**7.0** × **10**^−**15**^	−**0.045**	2.5 × 10^−3^	−0.015	—	—
rs137854526‡	C/T	0.24	*ANO5*	**4.5** × **10**^−**4**^	**0.204**	**1.1** × **10**^−**3**^	**0.190**	0.25	0.058	—	—
chr11:22241070‡	A/T	0.27	*ANO5*	**3.9** × **10**^−**6**^	**0.245**	**1.7** × **10**^−**5**^	**0.229**	0.05	0.09	—	—
rs2930191	A/G	37.22	—	**2.3** × **10**^−**4**^	−0.021	—	—	**4.7** × **10**^−**13**^	−0.035	—	—
chr12:7282745	TA/!TA	21.13	—	0.49	−0.005	—	—	**1.2** × **10**^−**25**^	−**0.064**	**2.3** × **10**^−**12**^	−**0.043**
rs145411783‡	A/C	0.66	*CD163L1*	0.90	0.005	—	—	**8.7** × **10**^−**12**^	**0.203**	**8.9** × **10**^−**10**^	**0.184**
rs4072797‡	T/C	4.21	*CD163L1*	0.63	0.007	—	—	**9.9** × **10**^−**89**^	−**0.236**	**5.5** × **10**^−**85**^	−**0.239**
rs117692263	C/T	9.31	*CD163*	**1.2** × **10**^−**17**^	**0.083**	**1.2** × **10**^−**11**^	**0.067**	**6.1** × **10**^**−28**^	**0.09**	**1.5** × **10**^−**13**^	**0.062**
rs4883263‡	T/C	3.73	*CD163*	**5.7** × **10**^−**5**^	0.059	—	—	**1.8** × **10**^**−19**^	**0.114**	**1.4** × **10**^−**6**^	**0.063**
rs7305678	T/G	16.16	—	**3.0** × **10**^−**21**^	**0.072**	**2.8** × **10**^−**15**^	**0.061**	**2.0** × **10**^−**18**^	**0.057**	**1.2** × **10**^−**23**^	**0.069**
chr12:110830276	C/T	2.09	—	**2.9** × **10**^−**4**^	−0.071	—	—	**2.1** × **10**^−**12**^	−**0.119**	—	—
rs7318906	A/G	47.08	—	**2.7** × **10**^−**13**^	−**0.041**	—	—	2.9 × 10^−3^	−0.014	—	—
rs149354459‡	G/C	0.21	*CKM*	**6.0** × **10**^−**20**^	−**0.55**	**2.8** × **10**^−**21**^	−**0.567**	0.54	0.031	—	—
rs145987658‡	A/G	0.086	*CKM*	**8.9** × **10**^−**9**^	−**0.59**	**2.1** × **10**^−**9**^	−**0.611**	0.58	0.047	—	—
rs17357122‡	A/G	0.99	*CKM*	**4.3** × **10**^−**9**^	−**0.165**	**4.3** × **10**^−**9**^	−**0.175**	0.45	0.018	—	—
rs11559024‡	C/T	2.15	*CKM*	**1.8** × **10**^−**115**^	−**0.446**	**1.1** × **10**^−**117**^	−**0.45**	0.16	0.023	—	—
rs393600	G/A	25.17	*LILRB5*	**1.4** × **10**^−**33**^	**0.079**	**1.3** × **10**^−**9**^	**0.046**	**1.6** × **10**^−**28**^	**0.062**	**1.7** × **10**^−**5**^	**0.028**
rs12975366‡	C/T	41.62	*LILRB5*	**6.5** × **10**^−**44**^	−**0.08**	**4.7** × **10**^−**20**^	−**0.061**	**7.0** × **10**^−**51**^	−**0.074**	**4.1** × **10**^−**28**^	−**0.062**

CK, creatine kinase; LDH, lactate dehydrogenase; MAF, minor allele frequency.

Variants showing significant association are in bold, based on the 25 variants tested (*P*<0.05/25=2 × 10^−3^). Further information can be found in Supplementary Table 1.

^*^Genes given for intronic/exonic variants.

^†^All variants are adjusted for all other significant variants in the same megabase.

^‡^Non-synonymous coding variants.

## References

[b1] Wallimann T., Wyss M., Brdiczka D., Nicolay K. & Eppenberger H. M. Intracellular compartmentation, structure and function of creatine kinase isoenzymes in tissues with high and fluctuating energy demands: the 'phosphocreatine circuit' for cellular energy homeostasis. Biochem. J. 281, 21–40 (1992). 1731757 10.1042/bj2810021 PMC1130636

[b2] Jacobson T. A. Toward "pain-free" statin prescribing: clinical algorithm for diagnosis and management of myalgia. Mayo Clin. Proc. 83, 687–700 (2008). 18533086 10.4065/83.6.687

[b3] Jacobson T. A. NLA Task Force on Statin Safety – 2014 update. J. Clin. Lipidol. 8, S1–S4 (2014). 10.1016/j.jacl.2014.03.003 24793438

[b4] Bosch X., Poch E. & Grau J. M. Rhabdomyolysis and acute kidney injury. N. Engl. J. Med. 361, 62–72 (2009). 19571284 10.1056/NEJMra0801327

[b5] GTEx Portal. Version 4, Build #193. Avaiable at: http://www.gtexportal.org (Accessed on 3 February 2015).

[b6] Spriet L. L., Howlett R. A. & Heigenhauser G. J. An enzymatic approach to lactate production in human skeletal muscle during exercise. Med. Sci. Sports Exerc. 32, 756–763 (2000). 10776894 10.1097/00005768-200004000-00007

[b7] Jaffe A. S. . It's time for a change to a troponin standard. Circulation 102, 1216–1220 (2000). 10982533 10.1161/01.cir.102.11.1216

[b8] Xu R. Y., Zhu X. F., Yang Y. & Ye P. High-sensitive cardiac troponin T. J. Geriatr. Cardiol. 10, 102–109 (2013). 23610580 10.3969/j.issn.1671-5411.2013.01.015 PMC3627711

[b9] Lin J. P., Zheng G., Joo J. & Cupples L. A. Genome-wide linkage and association scans for quantitative trait loci of serum lactate dehydrogenase-the framingham heart study. Hum. Genomics Proteomics 2010, 905237 (2010). 20981236 10.4061/2010/905237 PMC2958689

[b10] Bathum L. . Evidence for a substantial genetic influence on biochemical liver function tests: results from a population-based Danish twin study. Clin. Chem. 47, 81–87 (2001). 11148181

[b11] Rapaport D., Colletto G. M. & Zatz M. Genetic and environmental components of serum creatine kinase (CK) and pyruvate kinase (PK) in normal twins: implication for genetic risks estimates in Duchenne muscular dystrophy carriers. Am. J. Med. Genet. 31, 291–298 (1988). 3232697 10.1002/ajmg.1320310206

[b12] Dube M. P. . CKM and LILRB5 are associated with serum levels of creatine kinase. Circ. Cardiovasc. Genet. 7, 880–886 (2014). 25214527 10.1161/CIRCGENETICS.113.000395

[b13] Kamatani Y. . Genome-wide association study of hematological and biochemical traits in a Japanese population. Nat. Genet. 42, 210–215 (2010). 20139978 10.1038/ng.531

[b14] Gudbjartsson D. F. . Large-scale whole-genome sequencing of the Icelandic population. Nat. Genet. 47, 435–444 (2015). 25807286 10.1038/ng.3247

[b15] Gudmundsson J. . Genetic correction of PSA values using sequence variants associated with PSA levels. Sci. Transl. Med. 2, 62ra92 (2010). 10.1126/scitranslmed.3001513 PMC3564581 21160077

[b16] Kong A. . Detection of sharing by descent, long-range phasing and haplotype imputation. Nat. Genet. 40, 1068–1075 (2008). 19165921 10.1038/ng.216 PMC4540081

[b17] Styrkarsdottir U. . Nonsense mutation in the LGR4 gene is associated with several human diseases and other traits. Nature 497, 517–520 (2013). 23644456 10.1038/nature12124

[b18] Sveinbjornsson G. . Rare mutations associating with serum creatinine and chronic kidney disease. Hum. Mol. Genet. 23, 6935–6943 (2014). 25082825 10.1093/hmg/ddu399

[b19] Eppenberger H. M., Dawson D. M. & Kaplan N. O. The comparative enzymology of creatine kinases. I. Isolation and characterization from chicken and rabbit tissues. J. Biol. Chem. 242, 204–209 (1967). 6016604

[b20] Cabaniss C. in Clinical Methods: The History, Physical, and Laboratory Examinations eds Walker H., Hall W., Hurst J. 161–163Butterworths (1990). 21250045

[b21] Le A. . Inhibition of lactate dehydrogenase A induces oxidative stress and inhibits tumor progression. Proc. Natl Acad. Sci. USA 107, 2037–2042 (2010). 20133848 10.1073/pnas.0914433107 PMC2836706

[b22] George S., Ishikawa Y., Perryman M. B. & Roberts R. Purification and characterization of naturally occurring and *in vitro* induced multiple forms of MM creatine kinase. J. Biol. Chem. 259, 2667–2674 (1984). 6698986

[b23] Wevers R. A., Delsing M., Klein Gebbink J. A. & Soons J. B. Post-synthetic changes in creatine kinase isozymes (EC 2.7.3.2). Clin. Chim. Acta 86, 323–327 (1978). 668123 10.1016/0009-8981(78)90388-1

[b24] Radi Z. A. . Increased serum enzyme levels associated with kupffer cell reduction with no signs of hepatic or skeletal muscle injury. Am. J. Pathol. 179, 240–247 (2011). 21703406 10.1016/j.ajpath.2011.03.029 PMC3123844

[b25] Moeller J. B. . CD163-L1 is an endocytic macrophage protein strongly regulated by mediators in the inflammatory response. J. Immunol. 188, 2399–2409 (2012). 22279103 10.4049/jimmunol.1103150

[b26] Politz O. . Stabilin-1 and -2 constitute a novel family of fasciclin-like hyaluronan receptor homologues. Biochem. J. 362, 155–164 (2002). 11829752 10.1042/0264-6021:3620155 PMC1222372

[b27] Cooper G. M. . Distribution and intensity of constraint in mammalian genomic sequence. Genome Res. 15, 901–913 (2005). 15965027 10.1101/gr.3577405 PMC1172034

[b28] Gronlund J., Vitved L., Lausen M., Skjodt K. & Holmskov U. Cloning of a novel scavenger receptor cysteine-rich type I transmembrane molecule (M160) expressed by human macrophages. J. Immunol. 165, 6406–6415 (2000). 11086079 10.4049/jimmunol.165.11.6406

[b29] Johnson A. D. . SNAP: a web-based tool for identification and annotation of proxy SNPs using HapMap. Bioinformatics 24, 2938–2939 (2008). 18974171 10.1093/bioinformatics/btn564 PMC2720775

[b30] Abecasis G. R. . An integrated map of genetic variation from 1,092 human genomes. Nature 491, 56–65 (2012). 23128226 10.1038/nature11632 PMC3498066

[b31] Maller J. . Common variation in three genes, including a noncoding variant in CFH, strongly influences risk of age-related macular degeneration. Nat. Genet. 38, 1055–1059 (2006). 16936732 10.1038/ng1873

[b32] Weber B. H. . The role of the complement system in age-related macular degeneration. Dtsch. Arztebl. Int. 111, 133–138 (2014). 24622760 10.3238/arztebl.2014.0133 PMC3959224

[b33] Waters A. M. & Licht C. aHUS caused by complement dysregulation: new therapies on the horizon. Pediatr. Nephrol. 26, 41–57 (2011). 20556434 10.1007/s00467-010-1556-4 PMC2991208

[b34] Schlaf G. . Constitutive expression and regulation of rat complement factor H in primary cultures of hepatocytes, Kupffer cells, and two hepatoma cell lines. Lab. Invest. 82, 183–192 (2002). 11850531 10.1038/labinvest.3780410

[b35] Deban L. . Binding of the long pentraxin PTX3 to factor H: interacting domains and function in the regulation of complement activation. J. Immunol. 181, 8433–8440 (2008). 19050261 10.4049/jimmunol.181.12.8433

[b36] Doni A. . Interactions of the humoral pattern recognition molecule PTX3 with the complement system. Immunobiology 217, 1122–1128 (2012). 22964239 10.1016/j.imbio.2012.07.004

[b37] Doni A. . An acidic microenvironment sets the humoral pattern recognition molecule PTX3 in a tissue repair mode. J. Exp. Med. 212, 905–925 (2015). 25964372 10.1084/jem.20141268 PMC4451130

[b38] Rodriguez de Cordoba S., Esparza-Gordillo J., Goicoechea de Jorge E., Lopez-Trascasa M. & Sanchez-Corral P. The human complement factor H: functional roles, genetic variations and disease associations. Mol. Immunol. 41, 355–367 (2004). 15163532 10.1016/j.molimm.2004.02.005

[b39] Barrow A. D. & Trowsdale J. The extended human leukocyte receptor complex: diverse ways of modulating immune responses. Immunol. Rev. 224, 98–123 (2008). 18759923 10.1111/j.1600-065X.2008.00653.x

[b40] Katz H. R. Inhibition of inflammatory responses by leukocyte Ig-like receptors. Adv. Immunol. 91, 251–272 (2006). 16938543 10.1016/S0065-2776(06)91007-4

[b41] de Bakker P. I. . A high-resolution HLA and SNP haplotype map for disease association studies in the extended human MHC. Nat. Genet. 38, 1166–1172 (2006). 16998491 10.1038/ng1885 PMC2670196

[b42] Bottazzo G. F., Pujol-Borrell R., Hanafusa T. & Feldmann M. Role of aberrant HLA-DR expression and antigen presentation in induction of endocrine autoimmunity. Lancet 2, 1115–1119 (1983). 6138647 10.1016/s0140-6736(83)90629-3

[b43] Sveinbjornsson G. . HLA class II sequence variants contribute to risk of tuberculosis in Caucasian populations. Nature Genetics in press (2015).

[b44] Zhou Z. & Jensen P. E. Structural characteristics of HLA-DQ that may impact DM editing and susceptibility to Type-1 diabetes. Front. Immunol. 4, 262 (2013). 24009614 10.3389/fimmu.2013.00262 PMC3756536

[b45] Yang H. . The association of HLA-DQA1*0401 and DQB1*0604 with thymomatous myasthenia gravis in northern Chinese patients. J. Neurol. Sci. 312, 57–61 (2012). 21917268 10.1016/j.jns.2011.08.023

[b46] Deitiker P. R. . Association of HLA Class II alleles and haplotypes with cervical dystonia: HLA DR13-DQ6 (DQB1*0604) homozygotes are at greatly increased risk of cervical dystonia in Caucasian Americans. Autoimmunity 44, 167–176 (2011). 20843162 10.3109/08916934.2010.509121

[b47] Ifergan I. . Role of Ninjurin-1 in the migration of myeloid cells to central nervous system inflammatory lesions. Ann. Neurol. 70, 751–763 (2011). 22162058 10.1002/ana.22519

[b48] Toyama T. . Ninjurin1 increases p21 expression and induces cellular senescence in human hepatoma cells. J. Hepatol. 41, 637–643 (2004). 15464245 10.1016/j.jhep.2004.06.027

[b49] Liewluck T. . ANO5-muscular dystrophy: clinical, pathological and molecular findings. Eur. J. Neurol. 20, 1383–1389 (2013). 23663589 10.1111/ene.12191

[b50] Pegoraro E. & Hoffman E. P. in GeneReviews(R) eds Pagon R. A.. University of Washington (1993).

[b51] van der Kooi A. J. . The heart in limb girdle muscular dystrophy. Heart 79, 73–77 (1998). 9505924 10.1136/hrt.79.1.73 PMC1728583

[b52] Wahbi K. . Dilated cardiomyopathy in patients with mutations in anoctamin 5. Int. J. Cardiol. 168, 76–79 (2013). 23041008 10.1016/j.ijcard.2012.09.070

[b53] Powers P. A., Liu S., Hogan K. & Gregg R. G. Molecular characterization of the gene encoding the gamma subunit of the human skeletal muscle 1,4-dihydropyridine-sensitive Ca2+ channel (CACNLG), cDNA sequence, gene structure, and chromosomal location. J. Biol. Chem. 268, 9275–9279 (1993). 8387489

[b54] Mahy B. W. & Rowson K. E. Isoenzymic specificity of impaired clearance in mice infected with Riley virus. Science 149, 756 (1965). 14325164 10.1126/science.149.3685.756

[b55] Smit M. J., Duursma A. M., Bouma J. M. & Gruber M. Receptor-mediated endocytosis of lactate dehydrogenase M4 by liver macrophages: a mechanism for elimination of enzymes from plasma. Evidence for competition by creatine kinase MM, adenylate kinase, malate, and alcohol dehydrogenase. J. Biol. Chem. 262, 13020–13026 (1987). 2820961

[b56] Hayashi T. . Enhanced clearance of lactic dehydrogenase-5 in severe combined immunodeficiency (SCID) mice: effect of lactic dehydrogenase virus on enzyme clearance. Int. J. Exp. Pathol. 73, 173–181 (1992). 1571277 PMC2002002

[b57] Bijsterbosch M. K. . Several dehydrogenases and kinases compete for endocytosis from plasma by rat tissues. Biochem. J. 229, 409–417 (1985). 2994634 10.1042/bj2290409 PMC1145073

[b58] Kzhyshkowska J., Gratchev A. & Goerdt S. Stabilin-1, a homeostatic scavenger receptor with multiple functions. J. Cell. Mol. Med. 10, 635–649 (2006). 16989725 10.1111/j.1582-4934.2006.tb00425.x PMC3933147

[b59] Mori Y. . Inhibitory immunoglobulin-like receptors LILRB and PIR-B negatively regulate osteoclast development. J. Immunol. 181, 4742–4751 (2008). 18802077 10.4049/jimmunol.181.7.4742

[b60] Brancaccio P., Maffulli N. & Limongelli F. M. Creatine kinase monitoring in sport medicine. Br. Med. Bull. 81–82, 209–230 (2007). 10.1093/bmb/ldm014 17569697

[b61] Devlin B. & Roeder K. Genomic control for association studies. Biometrics 55, 997–1004 (1999). 11315092 10.1111/j.0006-341x.1999.00997.x

