# Supplementary material for: Common and rare variants associating with serum levels of creatine kinase and lactate dehydrogenase
Source: Nat Commun. 2016 Feb 3;7:10572. doi: 10.1038/ncomms10572 (PMC4742860; doi:10.1038/ncomms10572)
Supplement: Supplementary Information — Supplementary Figures 1-35, Supplementary Tables 1-13, Supplementary Note 1 and Supplementary References [file ncomms10572-s1.pdf]

Supplementary Figures

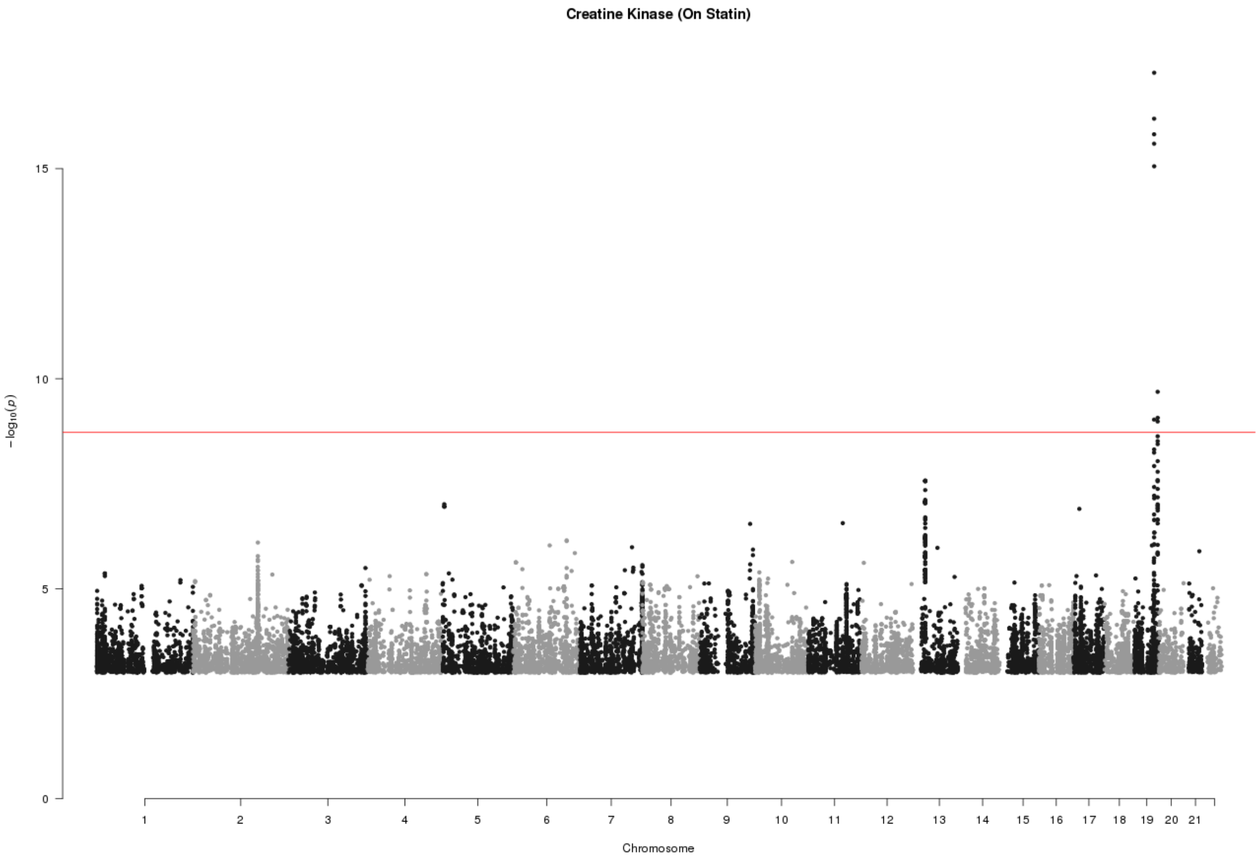

Supplementary Figure 1: *Manhattan plot showing the signals influencing serum creatine kinase (CK) levels in the Icelandic population during times of statin use. Variants are plotted by chromosomal position (x-axis) and -log10 P-values (y-axis). P-values above $1x10^{-25}$ are represented. Two loci (CKM* and *LILRB5) harbor variants with p-values below this cutoff. The red line indicates the threshold for genome-wide significance, determined by the number of tests performed (P = 0.05/28.3 million = $1.8x10^{-9}$).*

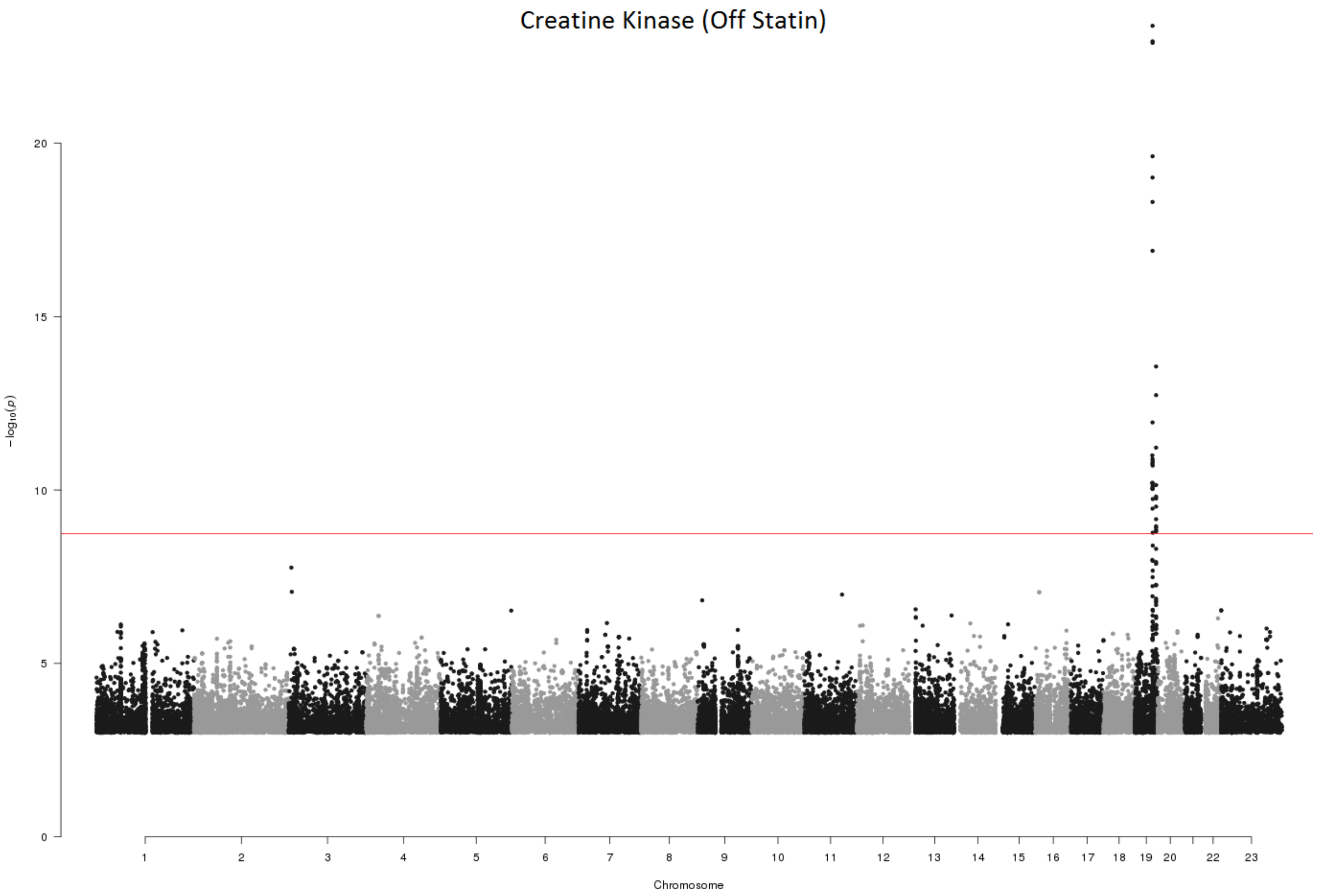

Supplementary Figure 2: *Manhattan plot showing the signals influencing serum creatine kinase (CK) levels in the Icelandic population outside of times of statin use. Variants are plotted by chromosomal position (x-axis) and -log10 P-values (y-axis). P-values above $1x10^{-25}$ are represented. Two loci (CKM* and *LILRB5) harbor variants with p-values below this cutoff. The red line indicates the threshold for genome-wide significance, determined by the number of tests performed (P = 0.05/28.3 million = $1.8x10^{-9}$).*

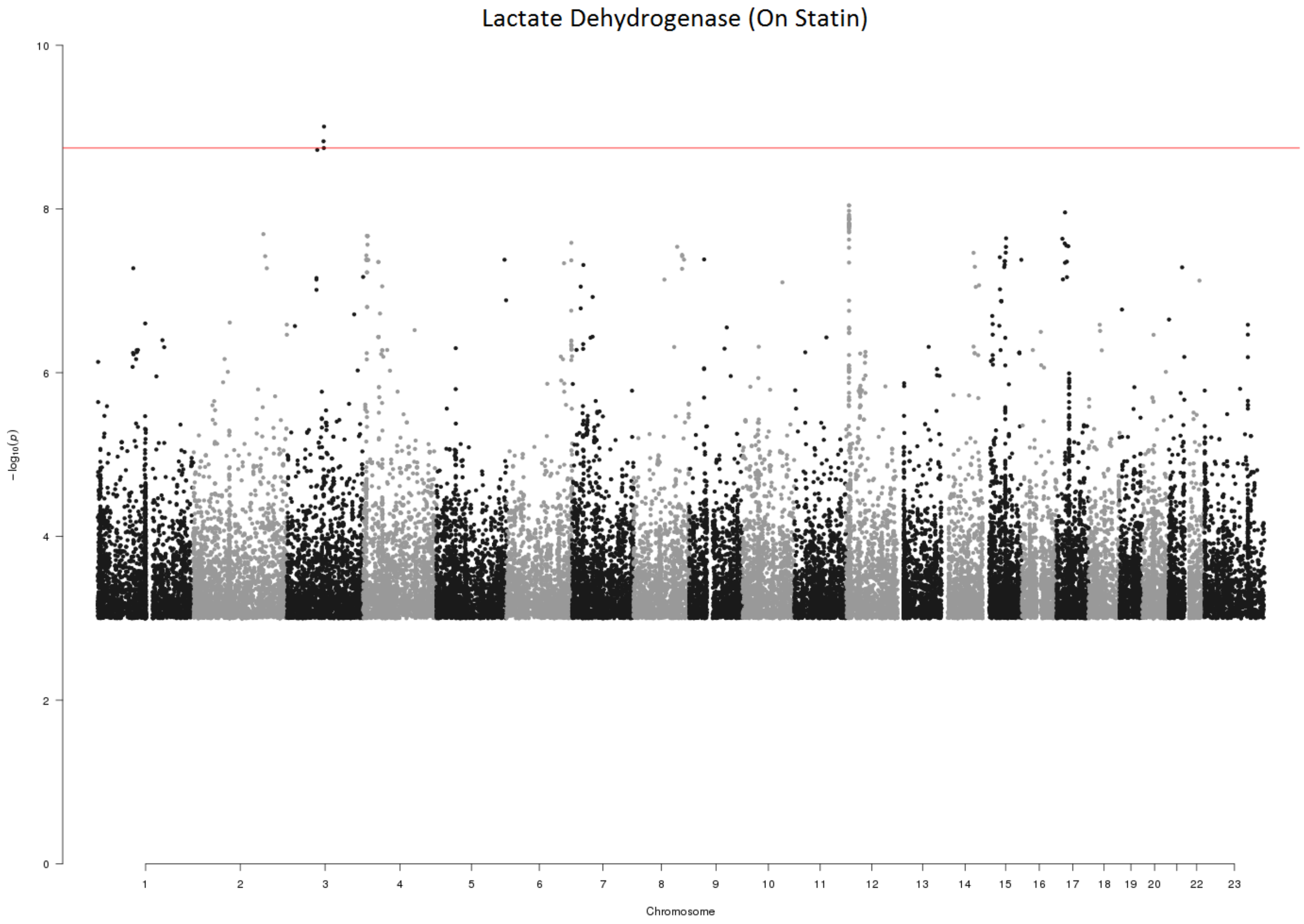

S**upplementary Figure 3:** *Manhattan plot showing the signals influencing serum lactate dehydrogenase (LDH) levels in the Icelandic population during times of statin use. Variants are plotted by chromosomal position (x-axis) and -log10 P-values (y-axis). P-values above* $1x10^{-25}$ *are represented. One locus (CD163L1) harbors variants with p-values below this cutoff. The red line indicates the threshold for genome-wide significance, determined by the number of tests performed (P = 0.05/28.3 million =* $1.8x10^{-9}$*).*

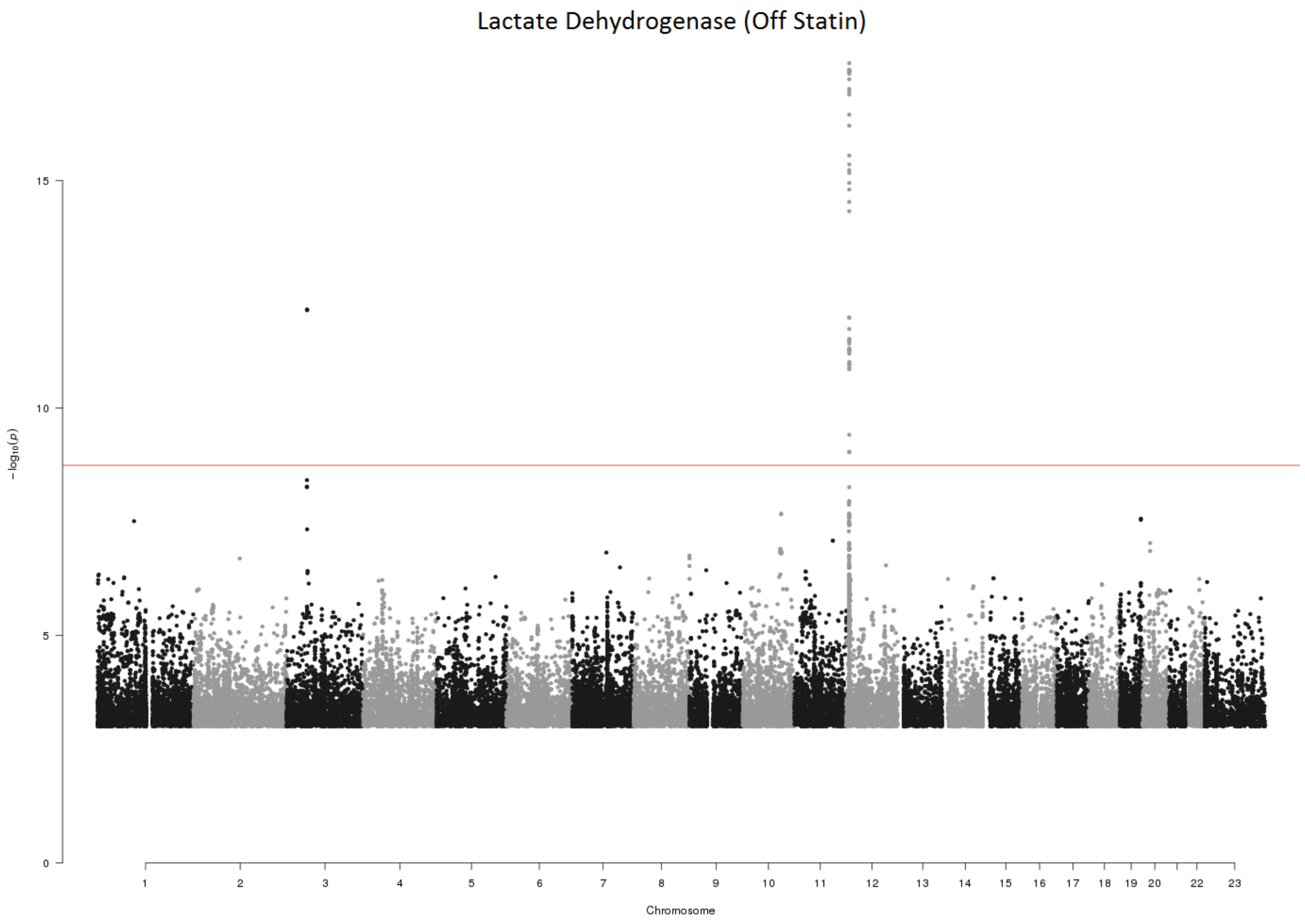

Supplementary Figure 4: *Manhattan plot showing the signals influencing serum lactate dehydrogenase (LDH) levels in the Icelandic population during times of statin use. Variants are plotted by chromosomal position (x-axis) and -log10 P-values (y-axis). P-values above $1x10^{-25}$ are represented. One locus (CD163L1) harbors variants with p-values below this cutoff. The red line indicates the threshold for genome-wide significance, determined by the number of tests performed (P = 0.05/28.3 million = $1.8x10^{-9}$).*

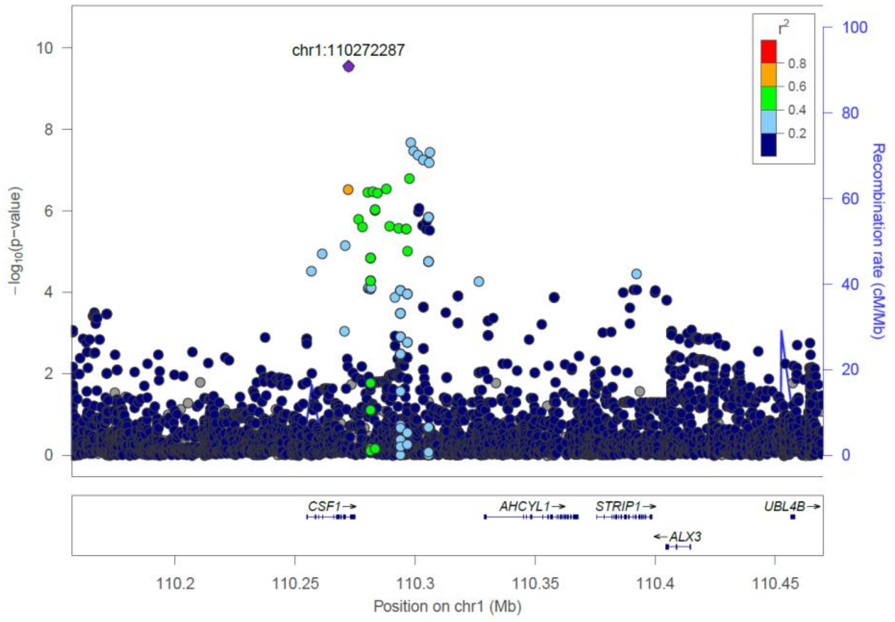

Supplementary Figure 5: *Locus plot depicting variants, arranged by chromosomal position (x-axis) within the CSF1 locus associating with serum LDH levels. The leading variant rs333947 is labelled and shown in purple, other variants are coloured according to correlation (r^2) with the leading marker (legend at top-right). –log_{10} P-values are shown along the left y-axis and correspond to the variants depicted in the plot. The right y-axis shows calculated recombination rates at the chromosomal location, plotted as a solid blue line.*

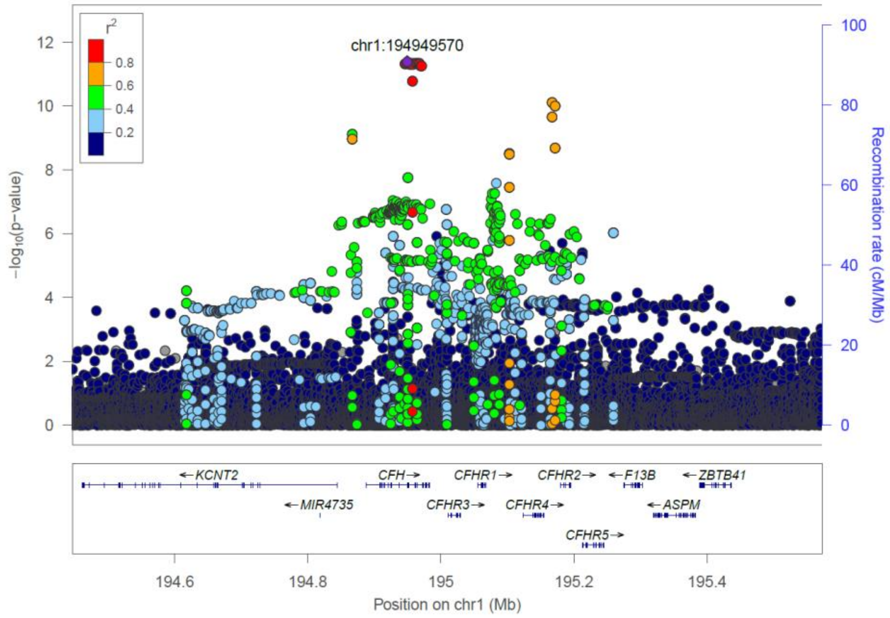

Supplementary Figure 6: *Locus plot depicting variants, arranged by chromosomal position (x-axis) within the CFH locus associating with serum LDH levels. The leading variant rs2274700 is labelled and shown in purple, other variants are coloured according to correlation (r^2) with the leading marker (legend at top-right). –log_{10} P-values are shown along the left y-axis and correspond to the variants depicted in the plot. The right y-axis shows calculated recombination rates at the chromosomal location, plotted as a solid blue line.*

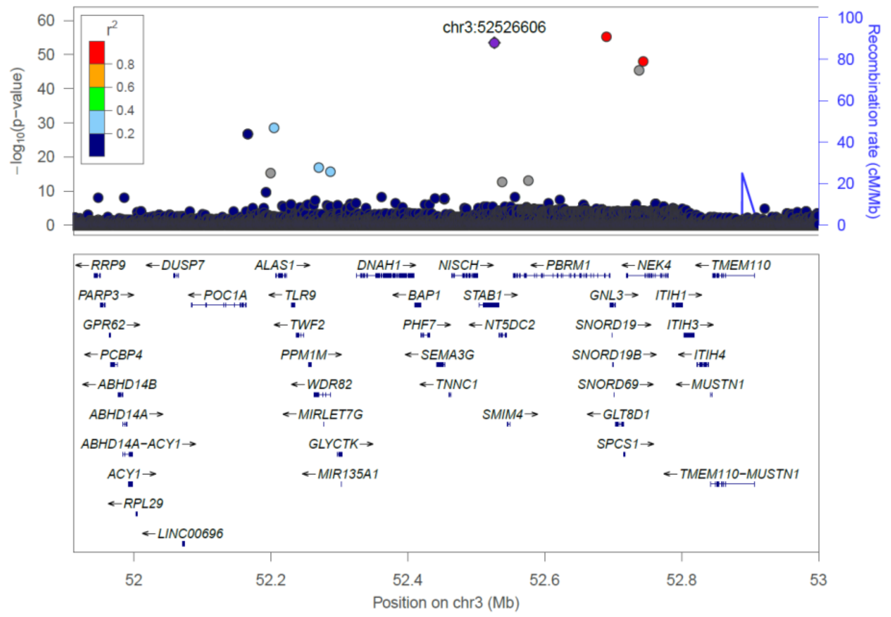

Supplementary Figure 7: *Locus plot depicting variants, arranged by chromosomal position (x-axis) within the STAB1 locus associating with serum LDH levels. The leading variant rs150956780 is labelled and shown in purple, other variants are coloured according to correlation (r^2) with the leading marker (legend at top-right). –log_{10} P-values are shown along the left y-axis and correspond to the variants depicted in the plot. The right y-axis shows calculated recombination rates at the chromosomal location, plotted as a solid blue line.*

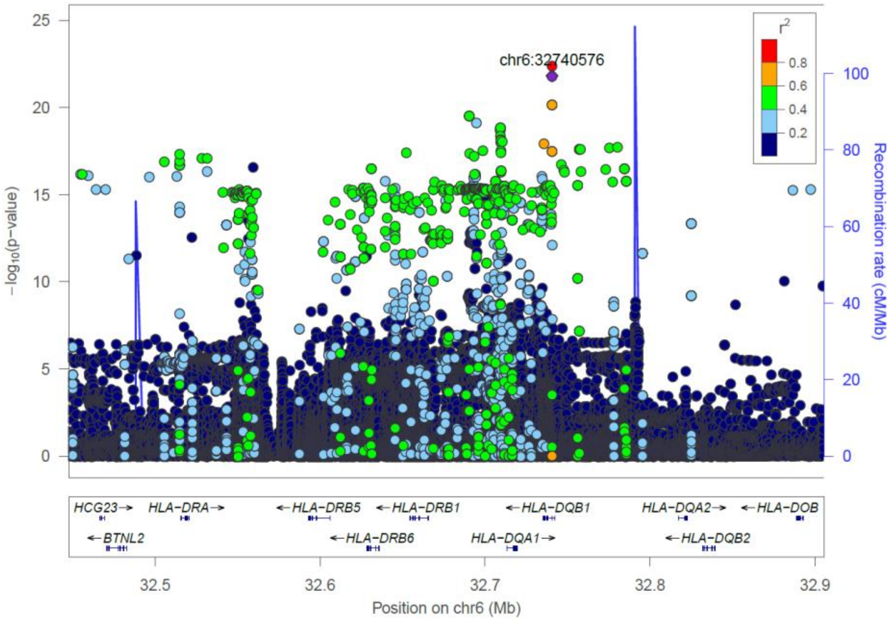

Supplementary Figure 8: *Locus plot depicting variants, arranged by chromosomal position (x-axis) within the HLA-DQB1 locus associating with serum LDH levels. The leading variant rs17412833 is labelled and shown in purple, other variants are coloured according to correlation (r^2) with the leading marker (legend at top-right). –log_{10} P-values are shown along the left y-axis and correspond to the variants depicted in the plot. The right y-axis shows calculated recombination rates at the chromosomal location, plotted as a solid blue line.*

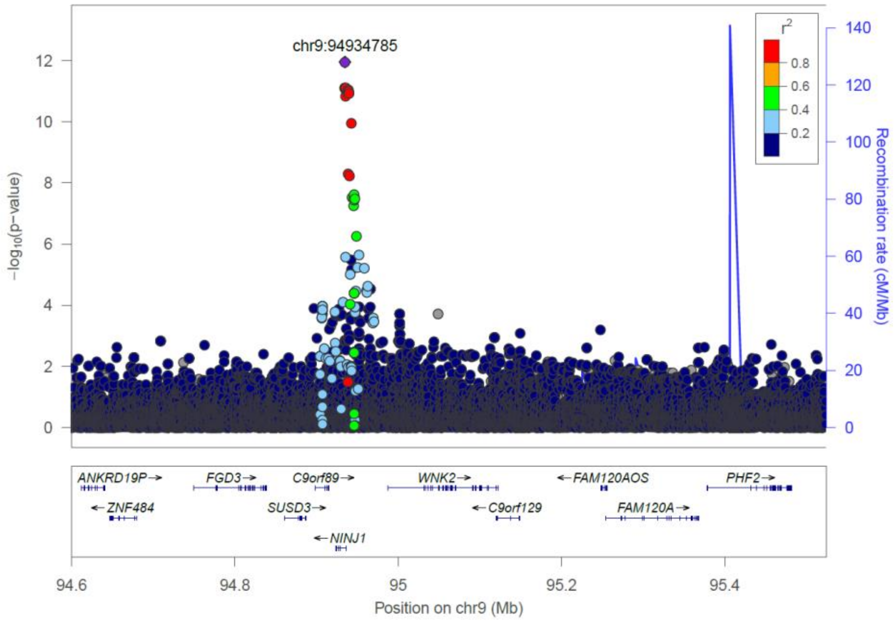

Supplementary Figure 9: *Locus plot depicting variants, arranged by chromosomal position (x-axis) within the NINJ1 locus associating with serum LDH levels. The leading variant rs12342201 is labelled and shown in purple, other variants are coloured according to correlation (r^2) with the leading marker (legend at top-right). –log_{10} P-values are shown along the left y-axis and correspond to the variants depicted in the plot. The right y-axis shows calculated recombination rates at the chromosomal location, plotted as a solid blue line.*

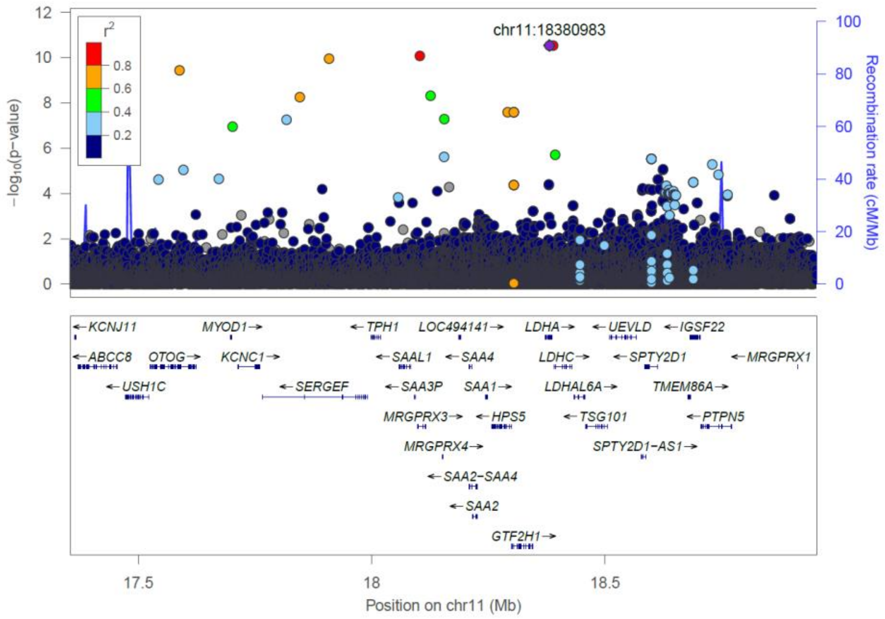

Supplementary Figure 10: *Locus plot depicting variants, arranged by chromosomal position (x-axis) within the LDHA locus associating with serum LDH levels. The leading variant rs116841148 is labelled and shown in purple, other variants are coloured according to correlation (r^2) with the leading marker (legend at top-right). –log_{10} P-values are shown along the left y-axis and correspond to the variants depicted in the plot. The right y-axis shows calculated recombination rates at the chromosomal location, plotted as a solid blue line.*

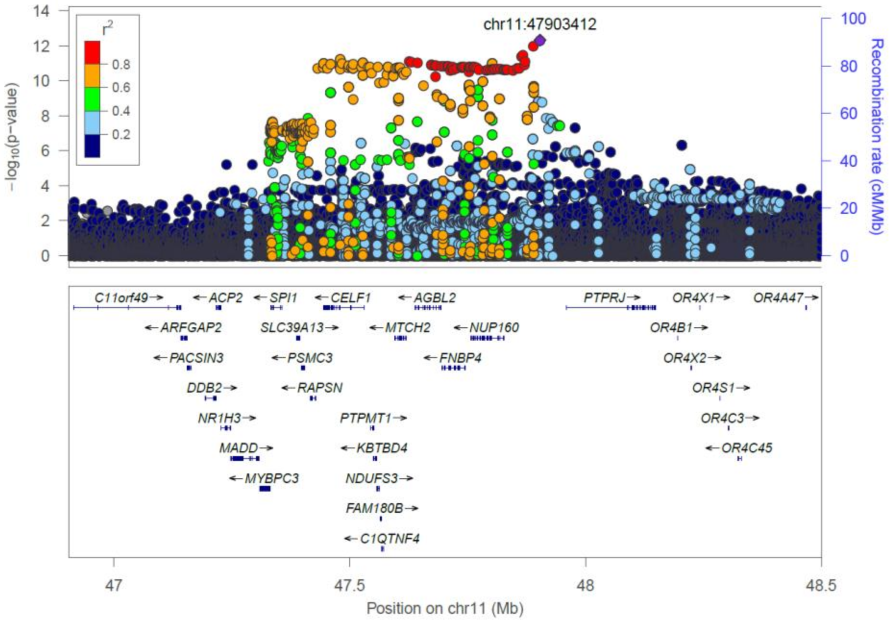

Supplementary Figure 11: *Locus plot depicting variants, arranged by chromosomal position (x-axis) at 11p11.2 associating with serum LDH levels. The leading variant chr11:47903412:S is labelled and shown in purple, other variants are coloured according to correlation (r^2) with the leading marker (legend at top-right). $-\log_{10}$ P-values are shown along the left y-axis and correspond to the variants depicted in the plot. The right y-axis shows calculated recombination rates at the chromosomal location, plotted as a solid blue line.*

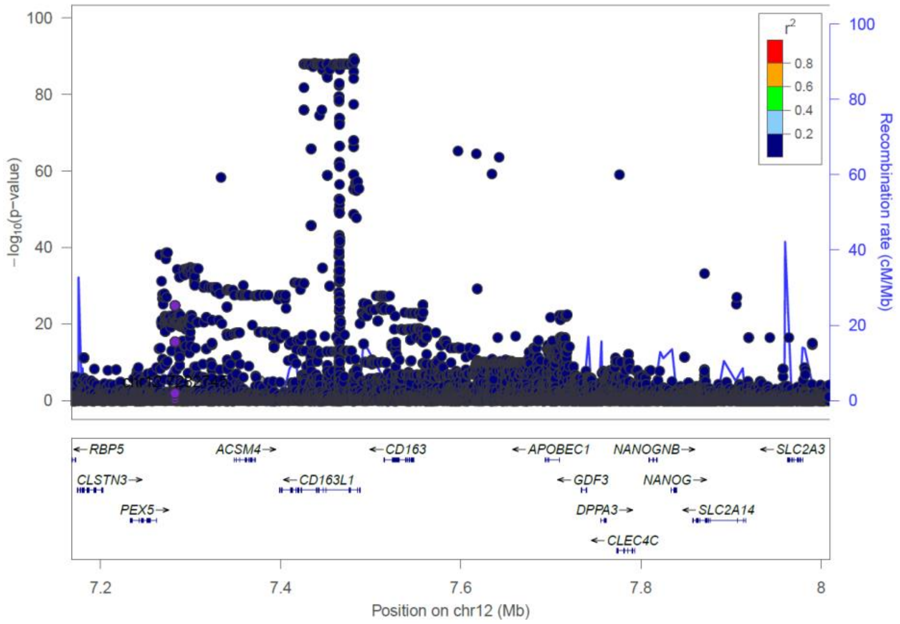

Supplementary Figure 12: *Locus plot depicting variants, arranged by chromosomal position (x-axis) at the CD163/CD163L1 locus associating with serum LDH levels. The leading variant chr12:7282745:0:TA is labelled and shown in purple, other variants are coloured according to correlation (r^2) with the leading marker (legend at top-right). $-\log_{10}$ P-values are shown along the left y-axis and correspond to the variants depicted in the plot. The right y-axis shows calculated recombination rates at the chromosomal location, plotted as a solid blue line.*

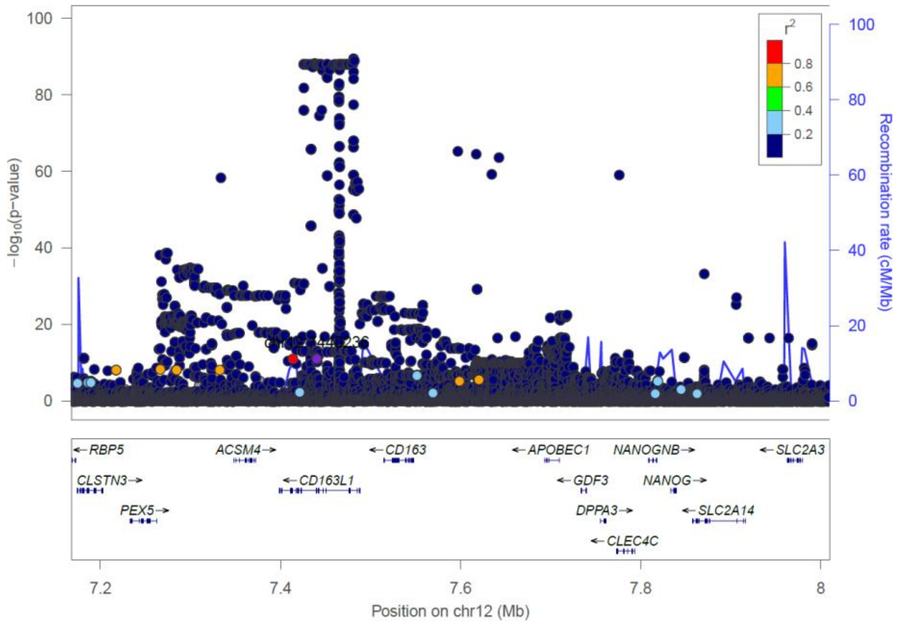

Supplementary Figure 13: *Locus plot depicting variants, arranged by chromosomal position (x-axis) at the CD163/CD163L1 locus associating with serum LDH levels. The leading variant rs145411783 is labelled and shown in purple, other variants are coloured according to correlation (r^2) with the leading marker (legend at top-right). –log_{10} P-values are shown along the left y-axis and correspond to the variants depicted in the plot. The right y-axis shows calculated recombination rates at the chromosomal location, plotted as a solid blue line.*

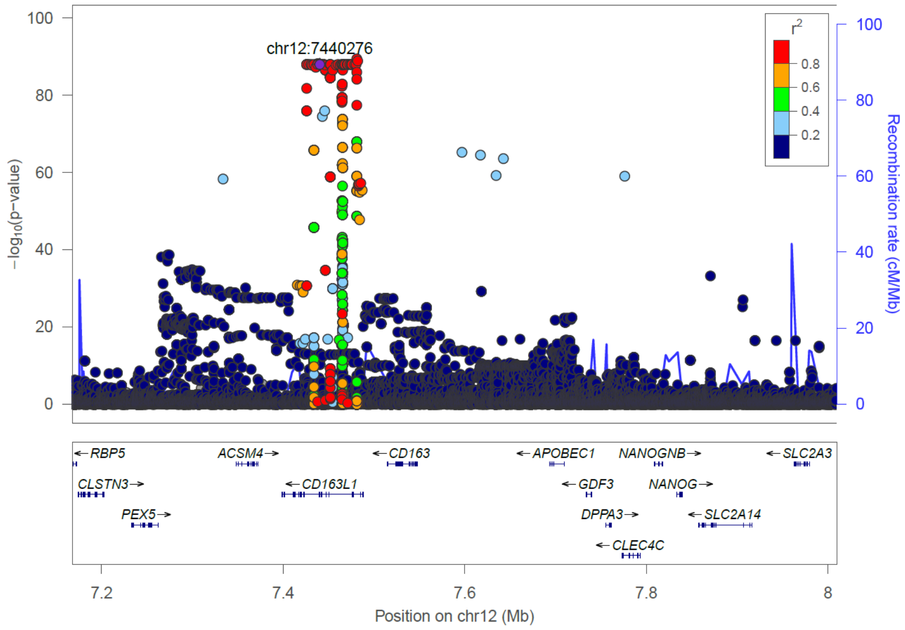

Supplementary Figure 14: *Locus plot depicting variants, arranged by chromosomal position (x-axis) at the CD163/CD163L1 locus associating with serum LDH levels. The leading variant rs4072797 is labelled and shown in purple, other variants are coloured according to correlation (r^2) with the leading marker (legend at top-right). –log_{10} P-values are shown along the left y-axis and correspond to the variants depicted in the plot. The right y-axis shows calculated recombination rates at the chromosomal location, plotted as a solid blue line.*

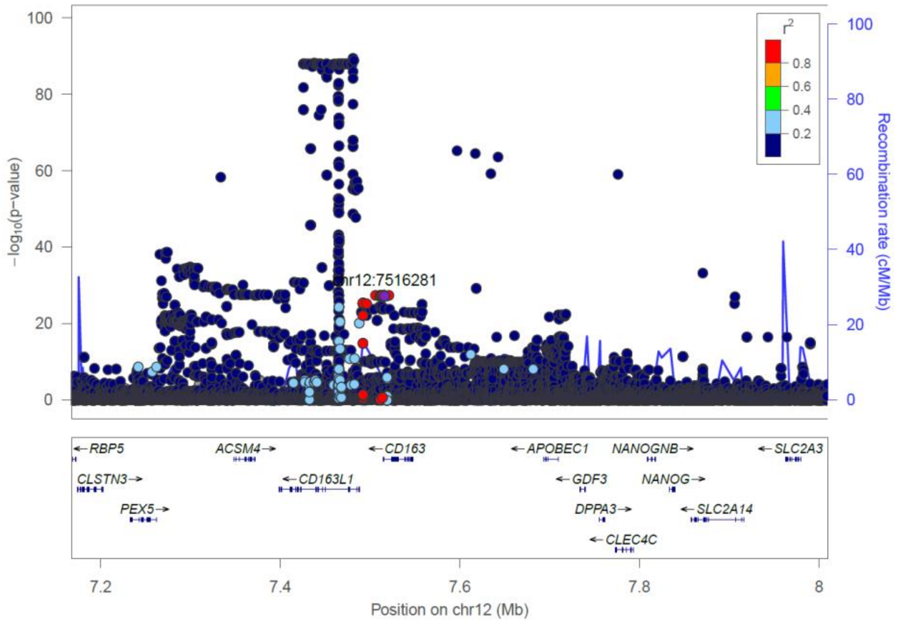

Supplementary Figure 15: *Locus plot depicting variants, arranged by chromosomal position (x-axis) at the CD163/CD163L1 locus associating with serum LDH levels. The leading variant rs117692263 is labelled and shown in purple, other variants are coloured according to correlation (r^2) with the leading marker (legend at top-right). –log_{10} P-values are shown along the left y-axis and correspond to the variants depicted in the plot. The right y-axis shows calculated recombination rates at the chromosomal location, plotted as a solid blue line.*

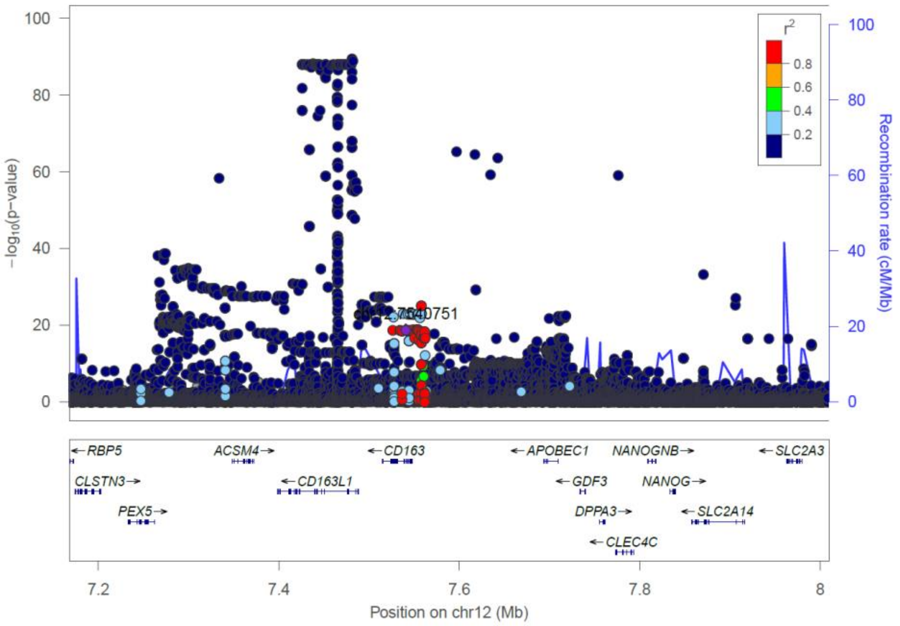

Supplementary Figure 16: *Locus plot depicting variants, arranged by chromosomal position (x-axis) at the CD163/CD163L1 locus associating with serum LDH levels. The leading variant rs4883263 is labelled and shown in purple, other variants are coloured according to correlation (r^2) with the leading marker (legend at top-right). –log_{10} P-values are shown along the left y-axis and correspond to the variants depicted in the plot. The right y-axis shows calculated recombination rates at the chromosomal location, plotted as a solid blue line.*

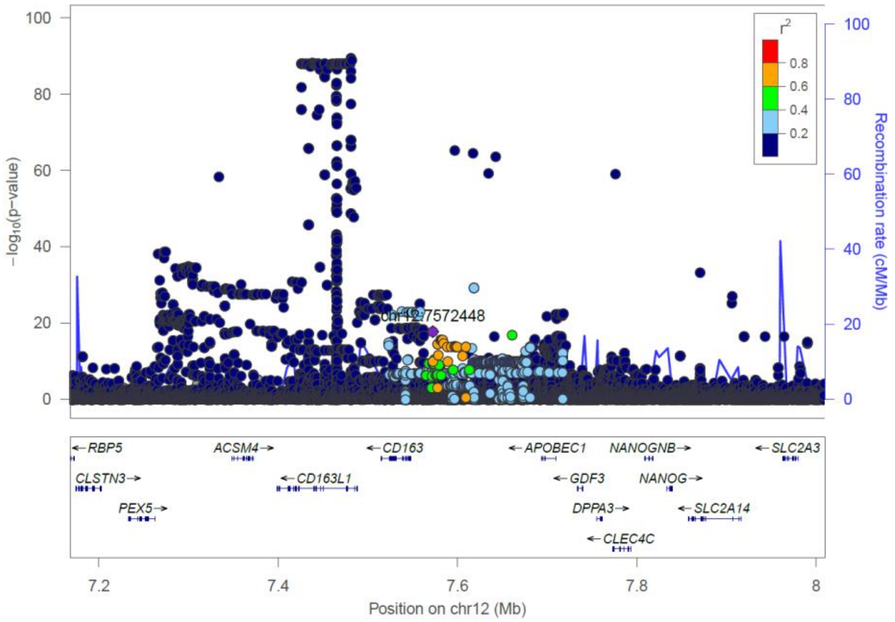

Supplementary Figure 17: *Locus plot depicting variants, arranged by chromosomal position (x-axis) at the CD163/CD163L1 locus associating with serum LDH levels. The leading variant rs7305678 is labelled and shown in purple, other variants are coloured according to correlation (r^2) with the leading marker (legend at top-right). –log_{10} P-values are shown along the left y-axis and correspond to the variants depicted in the plot. The right y-axis shows calculated recombination rates at the chromosomal location, plotted as a solid blue line.*

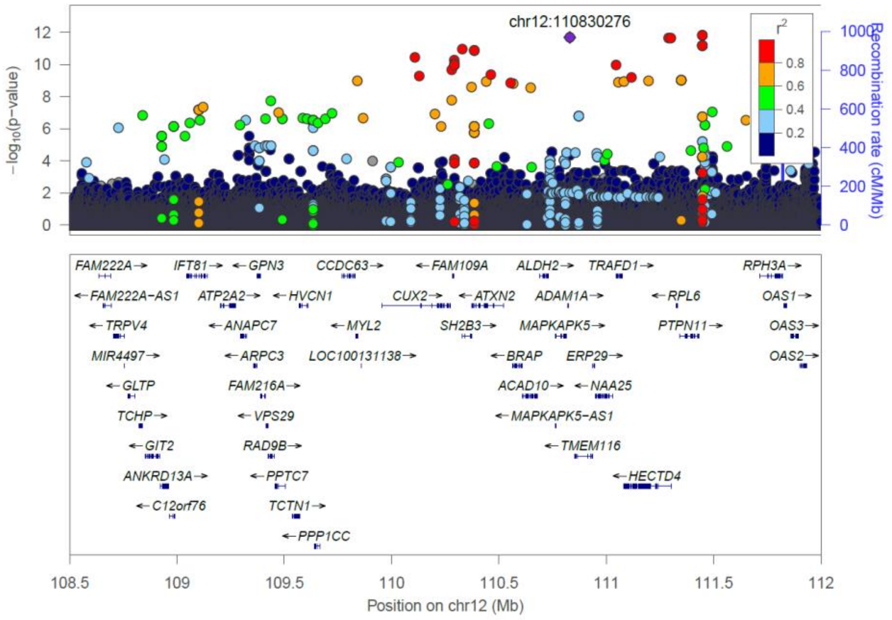

Supplementary Figure 18: *Locus plot depicting variants, arranged by chromosomal position (x-axis) at 12q24.13 associating with serum LDH levels. The leading variant chr12:110830276:S is labelled and shown in purple, other variants are coloured according to correlation (r^2) with the leading marker (legend at top-right). –log_{10} P-values are shown along the left y-axis and correspond to the variants depicted in the plot. The right y-axis shows calculated recombination rates at the chromosomal location, plotted as a solid blue line.*

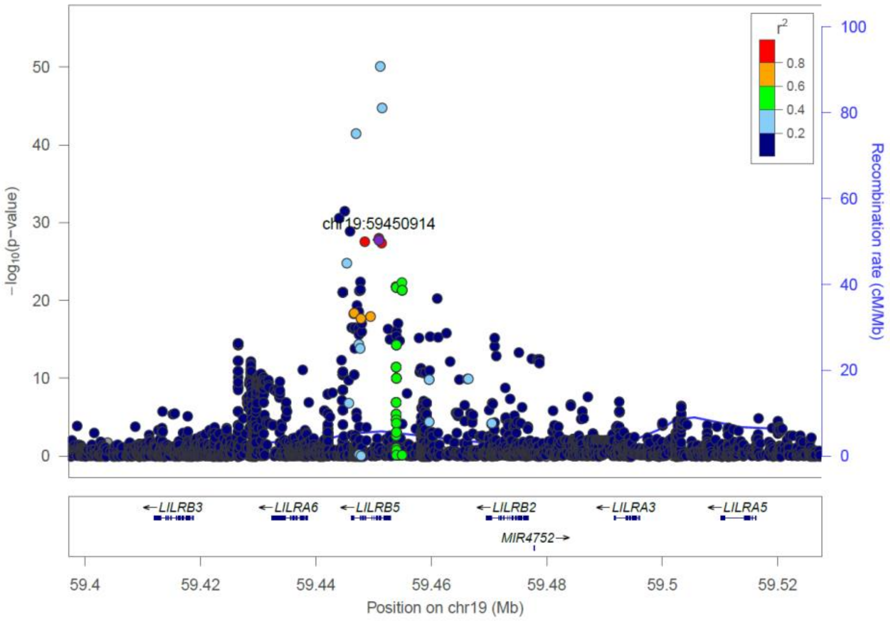

Supplementary Figure 19: *Locus plot depicting variants, arranged by chromosomal position (x-axis) at the LILRB5 locus associating with serum LDH levels. The leading variant rs393600 is labelled and shown in purple, other variants are coloured according to correlation (r^2) with the leading marker (legend at top-right). –log_{10} P-values are shown along the left y-axis and correspond to the variants depicted in the plot. The right y-axis shows calculated recombination rates at the chromosomal location, plotted as a solid blue line.*

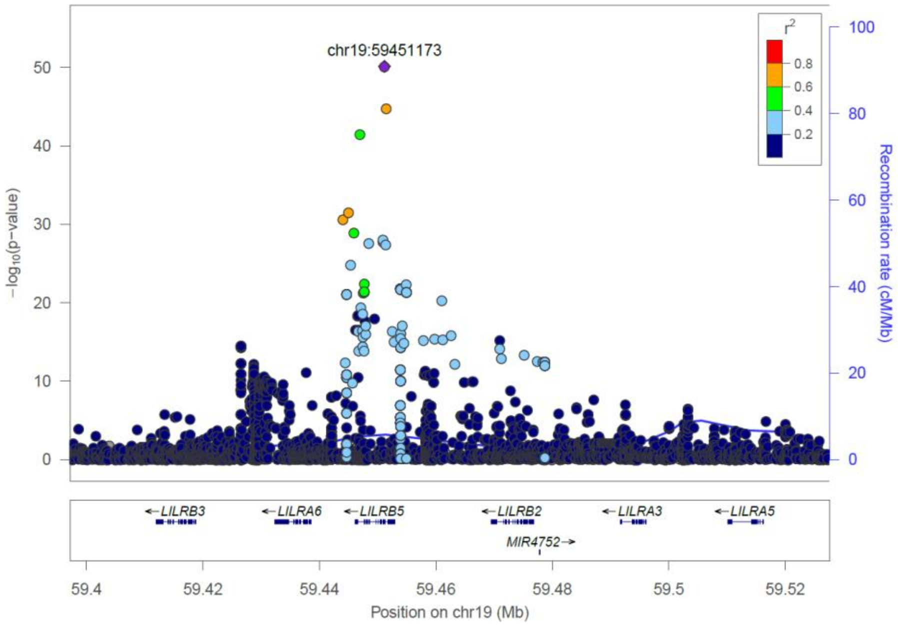

Supplementary Figure 20: *Locus plot depicting variants, arranged by chromosomal position (x-axis) at the LILRB5 locus associating with serum LDH levels. The leading variant rs12975366 is labelled and shown in purple, other variants are coloured according to correlation (r^2) with the leading marker (legend at top-right). –log_{10} P-values are shown along the left y-axis and correspond to the variants depicted in the plot. The right y-axis shows calculated recombination rates at the chromosomal location, plotted as a solid blue line.*

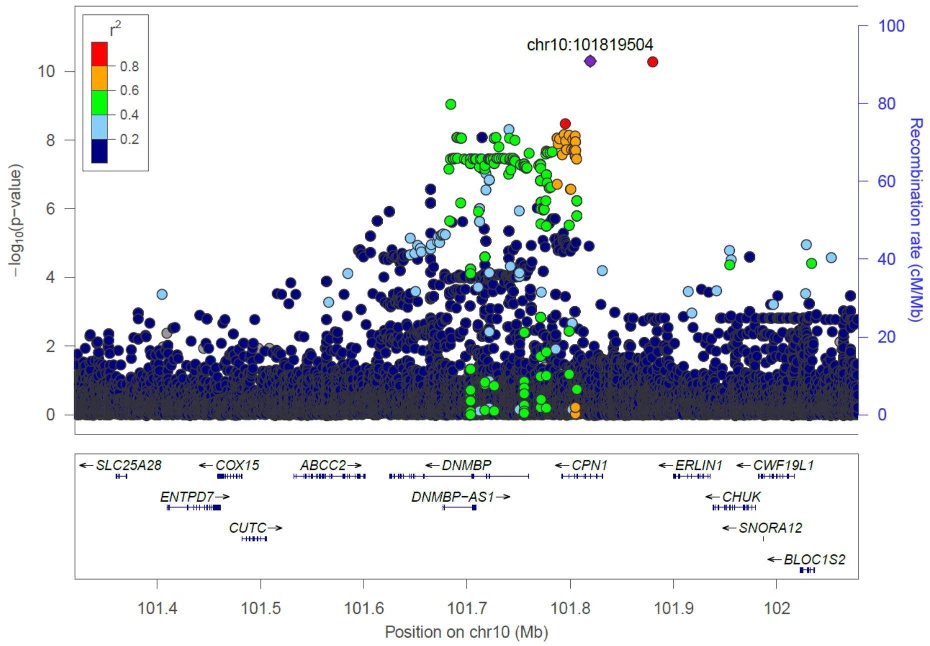

Supplementary Figure 21: *Locus plot depicting variants, arranged by chromosomal position (x-axis) at the CPN1 locus associating with serum CK levels. The leading variant rs61751507 is labelled and shown in purple, other variants are coloured according to correlation (r^2) with the leading marker (legend at top-right). –log_{10} P-values are shown along the left y-axis and correspond to the variants depicted in the plot. The right y-axis shows calculated recombination rates at the chromosomal location, plotted as a solid blue line.*

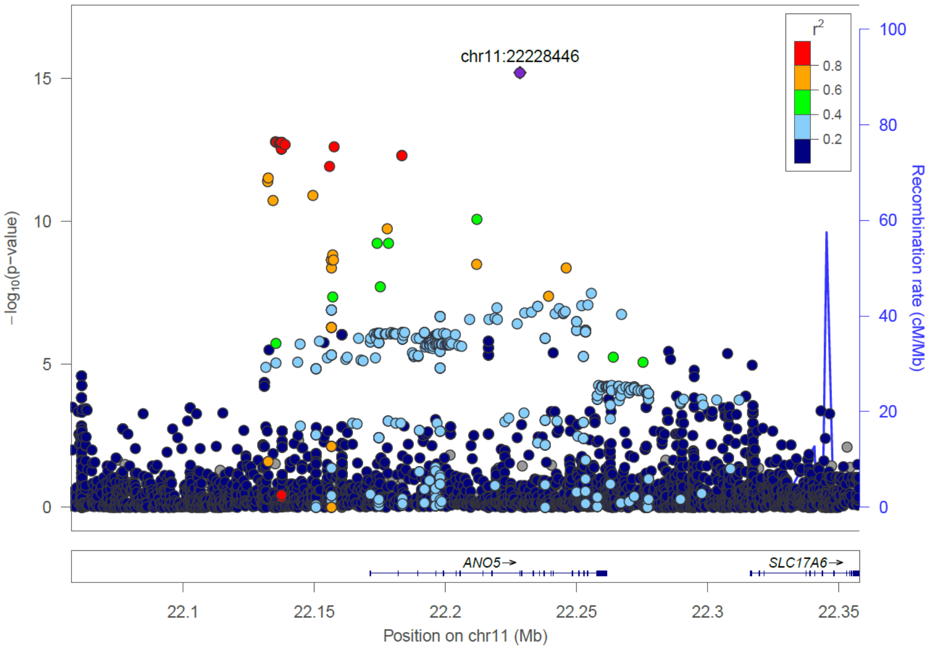

Supplementary Figure 22: *Locus plot depicting variants, arranged by chromosomal position (x-axis) at the ANO5 locus associating with serum CK levels. The leading variant rs7481951 is labelled and shown in purple, other variants are coloured according to correlation (r^2) with the leading marker (legend at top-right). –log_{10} P-values are shown along the left y-axis and correspond to the variants depicted in the plot. The right y-axis shows calculated recombination rates at the chromosomal location, plotted as a solid blue line.*

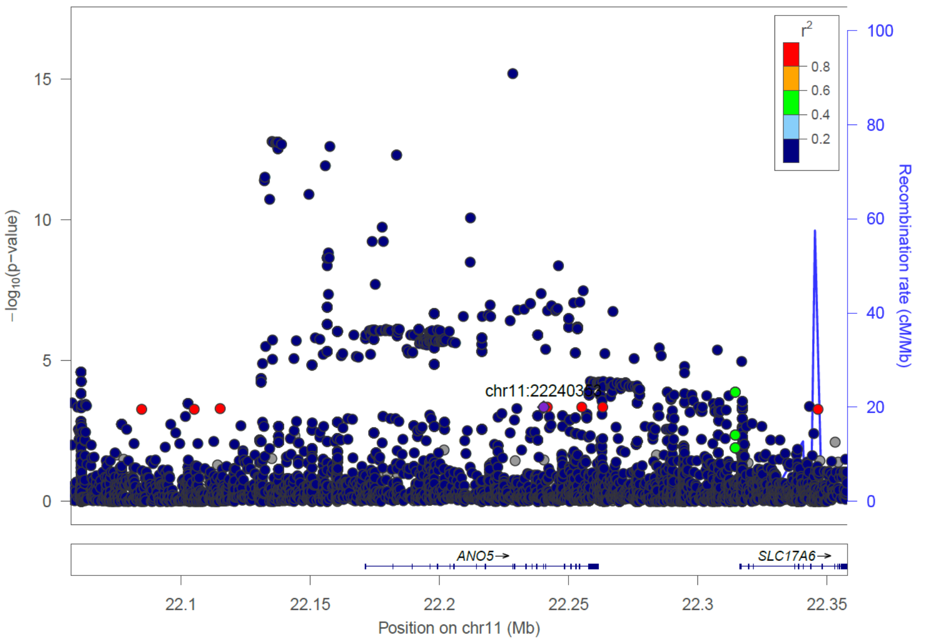

Supplementary Figure 23: *Locus plot depicting variants, arranged by chromosomal position (x-axis) at the ANO5 locus associating with serum CK levels. The leading variant rs137854526 is labelled and shown in purple, other variants are coloured according to correlation (r^2) with the leading marker (legend at top-right). –log_{10} P-values are shown along the left y-axis and correspond to the variants depicted in the plot. The right y-axis shows calculated recombination rates at the chromosomal location, plotted as a solid blue line.*

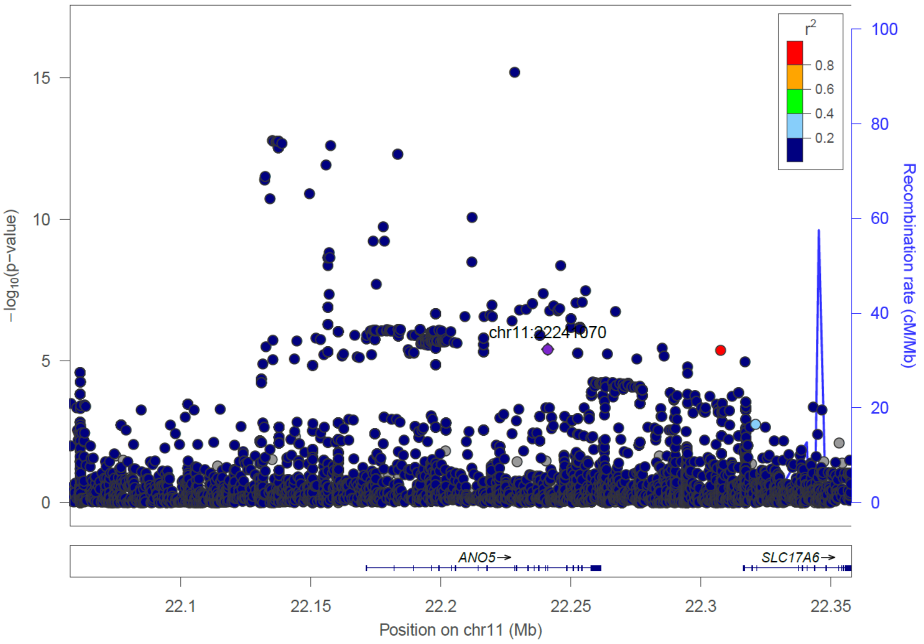

Supplementary Figure 24: *Locus plot depicting variants, arranged by chromosomal position (x-axis) at the ANO5 locus associating with serum CK levels. The leading variant chr11:22241070:S is labelled and shown in purple, other variants are coloured according to correlation (r^2) with the leading marker (legend at top-right). –log_{10} P-values are shown along the left y-axis and correspond to the variants depicted in the plot. The right y-axis shows calculated recombination rates at the chromosomal location, plotted as a solid blue line.*

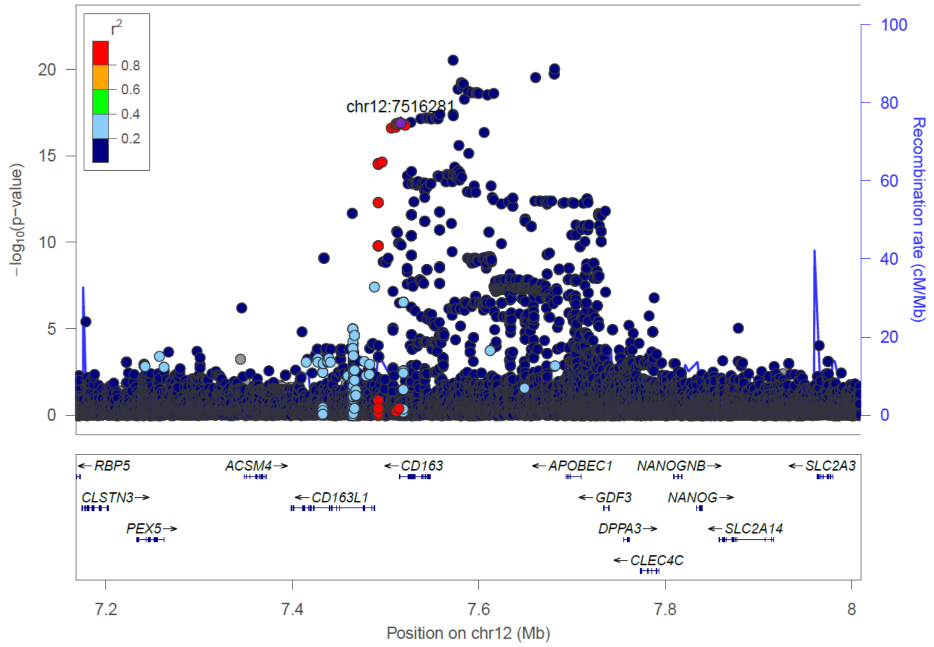

Supplementary Figure 25: *Locus plot depicting variants, arranged by chromosomal position (x-axis) at the CD163/CD163L1 locus associating with serum CK levels. The leading variant rs117692263 is labelled and shown in purple, other variants are coloured according to correlation (r^2) with the leading marker (legend at top-right). –log_{10} P-values are shown along the left y-axis and correspond to the variants depicted in the plot. The right y-axis shows calculated recombination rates at the chromosomal location, plotted as a solid blue line.*

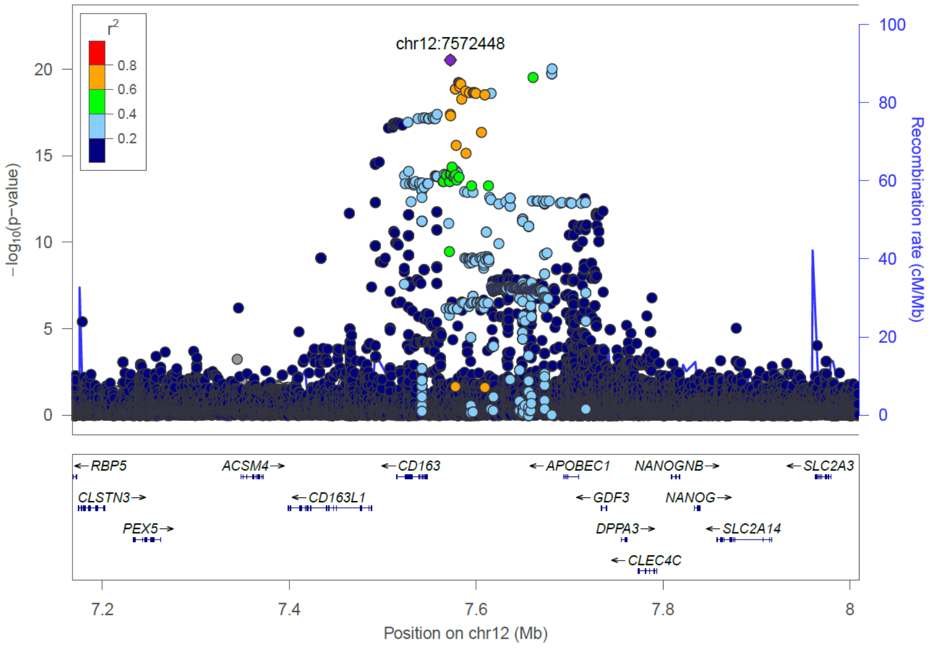

Supplementary Figure 26: *Locus plot depicting variants, arranged by chromosomal position (x-axis) at the CD163/CD163L1 locus associating with serum CK levels. The leading variant rs7305678 is labelled and shown in purple, other variants are coloured according to correlation (r^2) with the leading marker (legend at top-right). –log_{10} P-values are shown along the left y-axis and correspond to the variants depicted in the plot. The right y-axis shows calculated recombination rates at the chromosomal location, plotted as a solid blue line.*

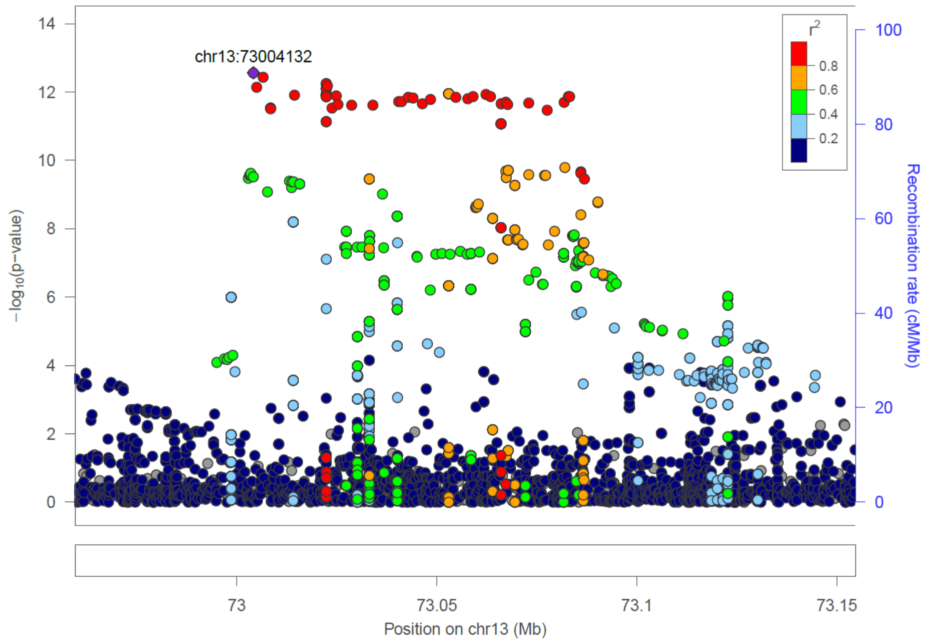

Supplementary Figure 27: *Locus plot depicting variants, arranged by chromosomal position (x-axis) at 13q22.1 associating with serum CK levels. The leading variant rs7318906 is labelled and shown in purple, other variants are coloured according to correlation (r^2) with the leading marker (legend at top-right). –log_{10} P-values are shown along the left y-axis and correspond to the variants depicted in the plot. The right y-axis shows calculated recombination rates at the chromosomal location, plotted as a solid blue line.*

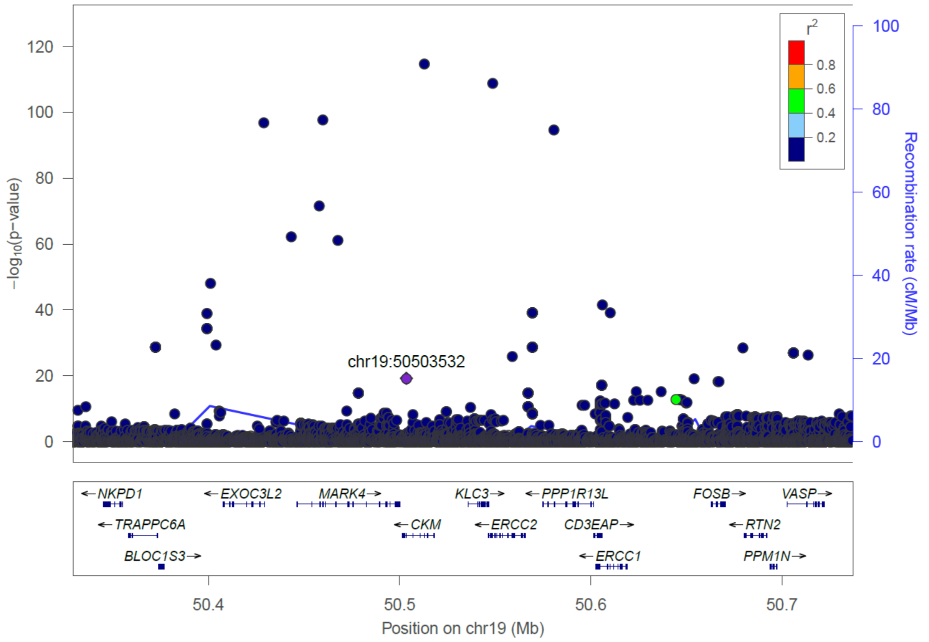

Supplementary Figure 28: *Locus plot depicting variants, arranged by chromosomal position (x-axis) at the CKM locus associating with serum CK levels. The leading variant rs149354459 is labelled and shown in purple, other variants are coloured according to correlation (r^2) with the leading marker (legend at top-right). –log_{10} P-values are shown along the left y-axis and correspond to the variants depicted in the plot. The right y-axis shows calculated recombination rates at the chromosomal location, plotted as a solid blue line.*

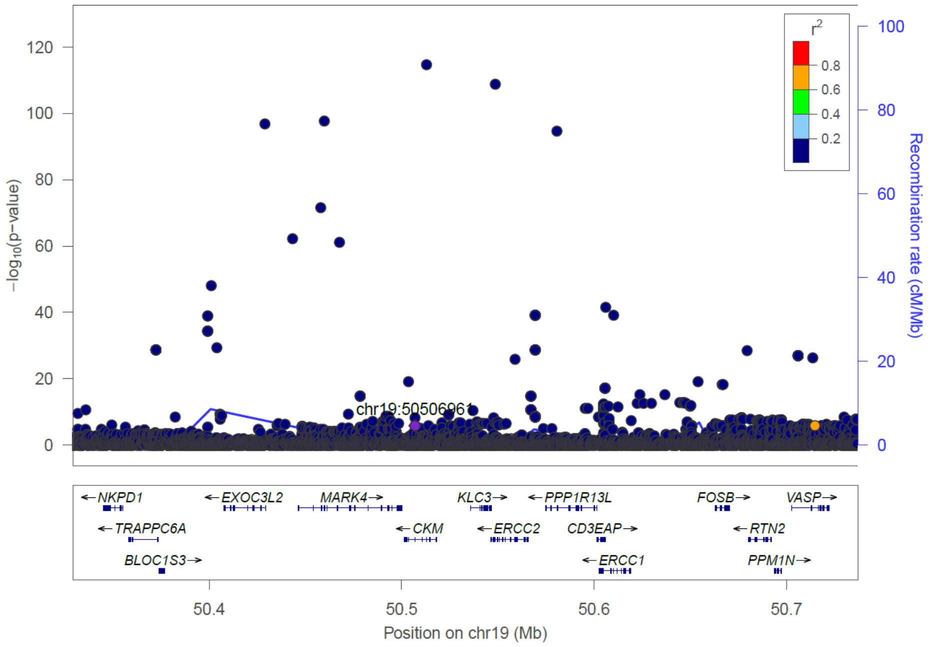

Supplementary Figure 29: *Locus plot depicting variants, arranged by chromosomal position (x-axis) at the CKM locus associating with serum CK levels. The leading variant rs145987658 is labelled and shown in purple, other variants are coloured according to correlation (r^2) with the leading marker (legend at top-right). –log_{10} P-values are shown along the left y-axis and correspond to the variants depicted in the plot. The right y-axis shows calculated recombination rates at the chromosomal location, plotted as a solid blue line.*

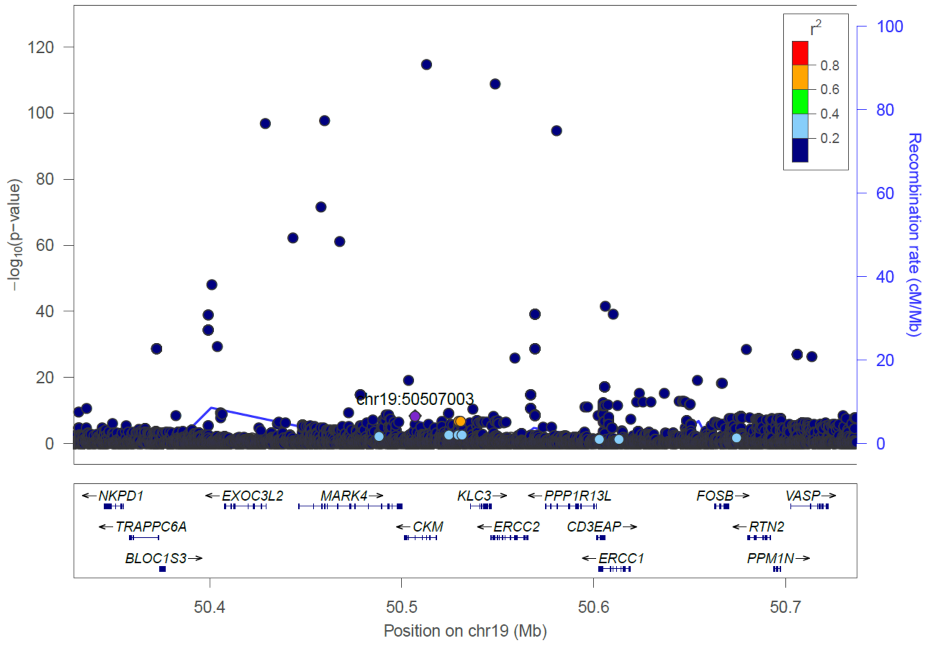

Supplementary Figure 30: *Locus plot depicting variants, arranged by chromosomal position (x-axis) at the CKM locus associating with serum CK levels. The leading variant rs17357122 is labelled and shown in purple, other variants are coloured according to correlation (r^2) with the leading marker (legend at top-right). –log_{10} P-values are shown along the left y-axis and correspond to the variants depicted in the plot. The right y-axis shows calculated recombination rates at the chromosomal location, plotted as a solid blue line.*

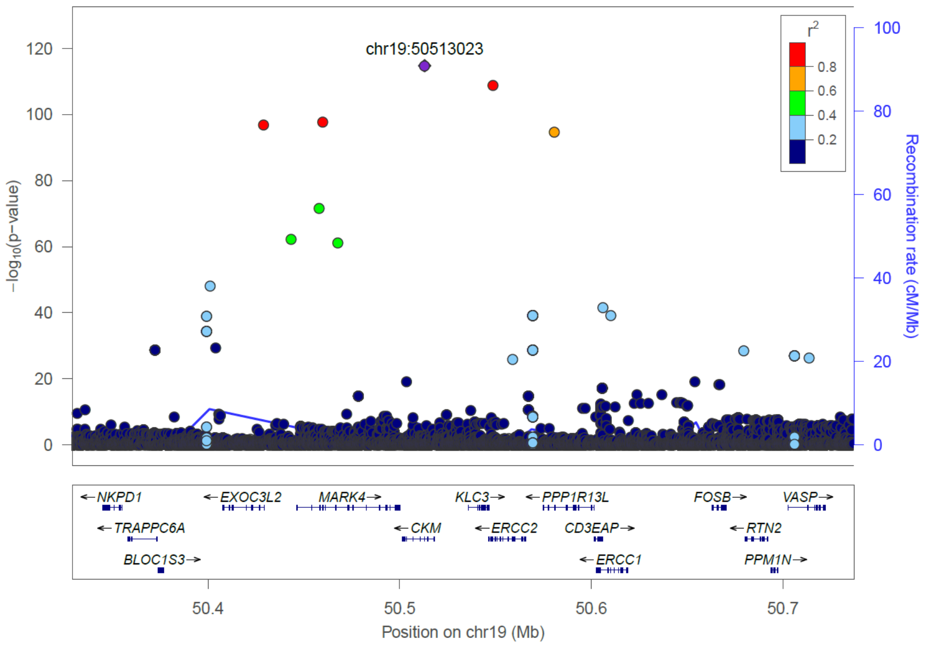

Supplementary Figure 31: *Locus plot depicting variants, arranged by chromosomal position (x-axis) at the CKM locus associating with serum CK levels. The leading variant rs11559024 is labelled and shown in purple, other variants are coloured according to correlation (r^2) with the leading marker (legend at top-right). $-log_{10}$ P-values are shown along the left y-axis and correspond to the variants depicted in the plot. The right y-axis shows calculated recombination rates at the chromosomal location, plotted as a solid blue line.*

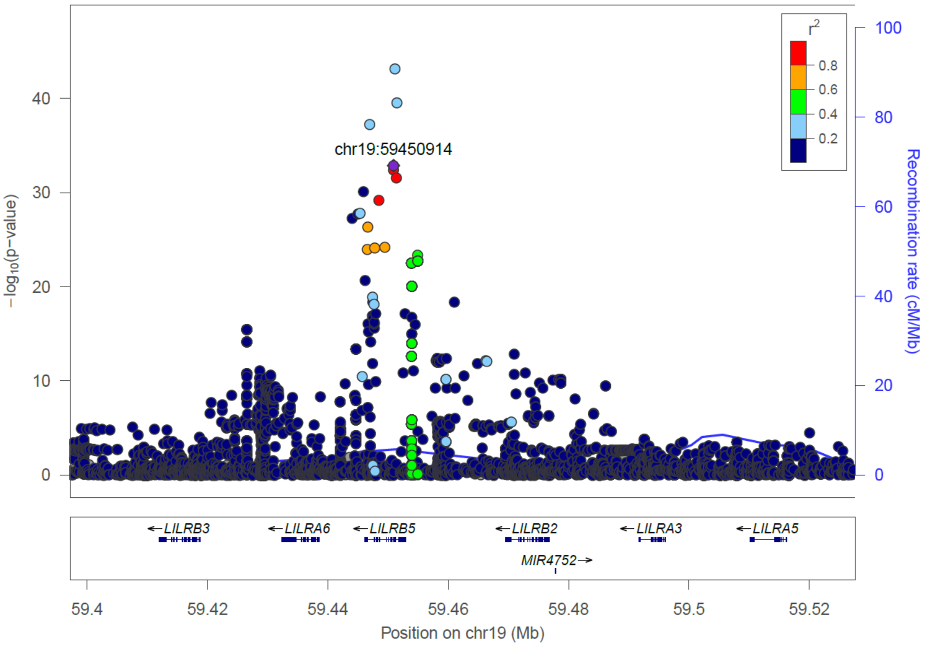

Supplementary Figure 32: *Locus plot depicting variants, arranged by chromosomal position (x-axis) at the LILRB5 locus associating with serum CK levels. The leading variant rs393600 is labelled and shown in purple, other variants are coloured according to correlation (r^2) with the leading marker (legend at top-right). $-log_{10}$ P-values are shown along the left y-axis and correspond to the variants depicted in the plot. The right y-axis shows calculated recombination rates at the chromosomal location, plotted as a solid blue line.*

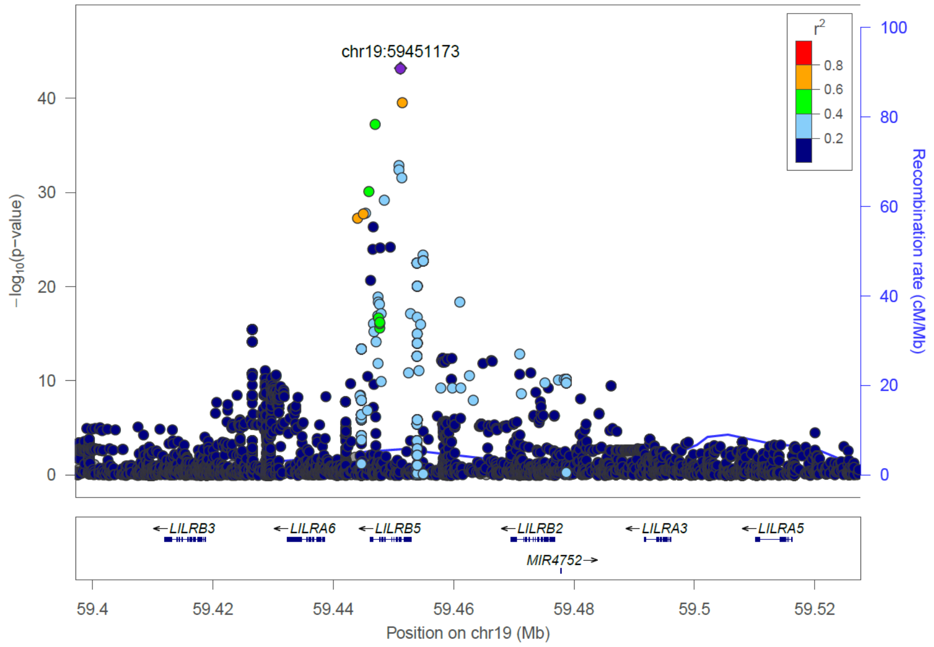

Supplementary Figure 33: *Locus plot depicting variants, arranged by chromosomal position (x-axis) at the LILRB5 locus associating with serum CK levels. The leading variant rs12975366 is labelled and shown in purple, other variants are coloured according to correlation (r^2) with the leading marker (legend at top-right). $–log_{10}$ P-values are shown along the left y-axis and correspond to the variants depicted in the plot. The right y-axis shows calculated recombination rates at the chromosomal location, plotted as a solid blue line.*

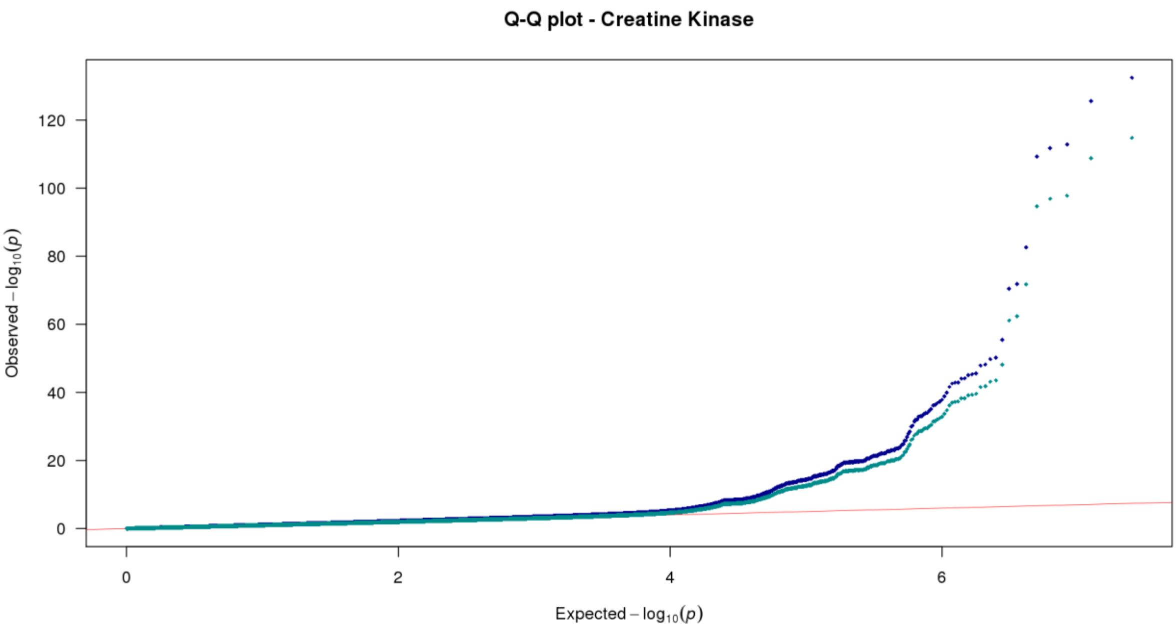

Supplementary Figure 34: *The plot shows uncorrected (dark blue) and corrected (cyan), using the method of genomic control, χ2 statistics from the creatine kinase GWAS.*

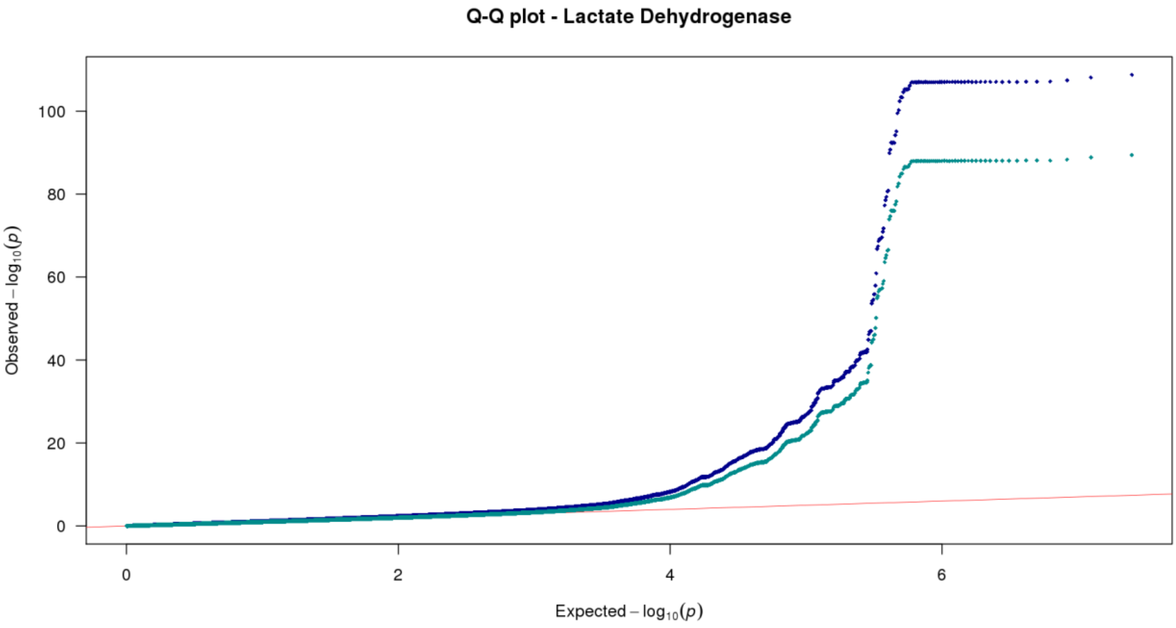

Supplementary Figure 35: *The plot shows uncorrected (dark blue) and corrected (cyan), using the method of genomic control, χ2 statistics from the lactate dehydrogenase GWAS*

Supplementary Tables

Supplementary Table 1: *Sequence variant coding effects and resulting amino acid changes to their protein transcripts*

Sequence Variant	Position (hg18)	Gene	Coding Effect	Coding Change
rs333947	chr1:110272287	*CSF1*	Intronic	-
rs2274700	chr1:194949570	*CFH*	Synonymous	NM_000186.3:c.1419G>A(p.=)
rs150956780	chr3:52526606	*STAB1*	Missense	NP_055951.2:p.Val1522Leu
rs17412833	chr6:32740576	*HLA-DQB1*	Missense	NP_001230890.1:p.Phe119Tyr, NP_002114.3:p.Phe119Tyr
rs12342201	chr9:94934785	*NINJ1*	Intronic	-
rs61751507	chr10:101819504	*CPN1*	Missense	NP_001299.1:p.Gly178Asp
rs116841148	chr11:18380983	*LDHA*	Missense	NP_001158886.1:p.Ala176Ser, NP_001158887.1:p.Ala147Ser
rs7481951	chr11:22228446	*ANO5*	Missense	NP_998764.1:p.Leu322Phe
rs137854526	chr11:22240353	*ANO5*	Missense	NP_998764.1:p.Phe578Ser
chr11:22241070:S	chr11:22241070	*ANO5*	Nonsense	NP_998764.1:p.Cys601X
rs2930191	chr11:47903412	-	-	-
chr12:7282745:0:TA	chr12:7282745	-	-	-
rs145411783	chr12:7440236	*CD163L1*	Missense	ENSP00000393474.2:p.Arg601Leu
rs4072797	chr12:7440276	*CD163L1*	Missense	ENSP00000393474.2:p.Asp588Asn
rs117692263	chr12:7516281	*CD163*	Intronic	-
rs4883263	chr12:7540751	*CD163*	Missense	NP_004235.4:p.Ile342Val, NP_981961.2:p.Ile342Val
rs7305678	chr12:7572448	-	-	-
chr12:110830276:S	chr12:110830276	-	-	-
rs7318906	chr13:73004132	-	-	-
rs149354459	chr19:50503532	*CKM*	Missense	NP_001815.2:p.Arg251Pro
rs145987658	chr19:50506961	*CKM*	Missense	NP_001815.2:p.Thr180Met
rs17357122	chr19:50507003	*CKM*	Missense	NP_001815.2:p.Thr166Met
rs11559024	chr19:50513023	*CKM*	Missense	NP_001815.2:p.Glu83Gly
rs393600	chr19:59450914	*LILRB5*	Intronic	-
rs12975366	chr19:59451173	*LILRB5*	Missense	NP_001074912.1:p.Asp147Gly, NP_006831.1:p.Asp247Gly

Supplementary Table 2: *Associations between CK and all reported variants when testing alternative inheritance models*

							Additive		Dominant		Recessive	
**Variant**	**Chr**	**Position (hg18)**	**Allele (min/maj)**	**MAF (%)**	**Gene[††]**	**Coding Change**	***P*-value**	**Effect [Amin]**	***P*-value**	**Effect [Amin]**	***P*-value**	**Effect [Amin]**
rs333947	1	110,272,287	A/G	14.92	*CSF1*	-	6.8×10^{-3}	-0.021	2.03×10^{-3}	-0.027	0.97	-0.001
rs2274700	1	194,949,570	A/G	38.62	*CFH*	-	0.58	-0.003	0.71	-0.003	0.54	-0.006
rs150956780	3	52,526,606	C/G	0.078	*STAB1*	Val1522Leu	0.60	0.05	0.40	0.132	na	na
rs17412833	6	32,740,576	T/A	13.76	*HLA-DQB1*	Phe119Tyr	1.5×10^{-4}	0.032	4.31×10^{-4}	0.033	5.63×10^{-3}	0.08
rs12342201	9	94,934,785	A/G	49.45	*NINJ1*	-	0.70	0.002	0.88	-0.001	0.41	0.007
rs61751507	10	101,819,504	T/C	4.06	*CPN1*	Gly178Asp	5.1×10^{-11}	-0.091	9.92×10^{-12}	-0.097	0.47	-0.063
rs116841148	11	18,380,983	T/G	0.65	*LDHA*	Ala176Ser, Ala147Ser	0.17	0.049	0.16	0.05	na	na
rs7481951	11	22,228,446	A/T	37.45	*ANO5*	Leu322Phe	6.4×10^{-16}	-0.047	7.32×10^{-16}	-0.065	2.68×10^{-7}	-0.056
rs137854526	11	22,240,353	C/T	0.24	*ANO5*	Phe578Ser	4.5×10^{-4}	0.204	5.09×10^{-4}	0.201	0.11	1.229
chr11:22241070	11	22,241,070	A/T	0.27	*ANO5*	Cys601X	3.9×10^{-6}	0.245	2.71×10^{-6}	0.246	na	na
rs2930191	11	47,903,412	A/G	37.22	-	-	2.3×10^{-4}	-0.021	1.80×10^{-3}	-0.025	1.58×10^{-3}	-0.035
chr12:7282745	12	7,282,745	TA/!TA	21.13	-	-	0.49	-0.005	0.46	-0.006	0.81	-0.005
rs145411783	12	7,440,236	A/C	0.66	*CD163L1*	Arg601Leu	0.90	0.005	0.81	0.008	0.13	-0.706
rs4072797	12	7,440,276	T/C	4.21	*CD163L1*	Asp588Asn	0.63	0.007	0.66	0.006	0.65	0.041
rs117692263	12	7,516,281	C/T	9.31	*CD163*	-	1.2×10^{-17}	0.083	1.06×10^{-16}	0.085	3.68×10^{-6}	0.194
rs4883263	12	7,540,751	T/C	3.73	*CD163*	Ile342Val	5.7×10^{-5}	0.059	4.15×10^{-5}	0.061	0.40	0.085
rs7305678	12	7,572,448	T/G	16.16	-	-	3.0×10^{-21}	0.072	1.14×10^{-19}	0.078	8.21×10^{-9}	0.137
chr12:110830276	12	110,830,276	C/T	2.09	-	-	2.9×10^{-4}	-0.071	1.65×10^{-4}	-0.074	0.79	0.05
rs7318906	13	73,004,132	A/G	47.08	-	-	2.7×10^{-13}	-0.041	7.10×10^{-11}	-0.057	3.83×10^{-9}	-0.053
rs149354459	19	50,503,532	G/C	0.21	*CKM*	Arg251Pro	6.0×10^{-20}	-0.55	1.60×10^{-20}	-0.552	na	na
rs145987658	19	50,506,961	A/G	0.086	*CKM*	Thr180Met	8.9×10^{-9}	-0.59	8.06×10^{-7}	-0.425	na	na
rs17357122	19	50,507,003	A/G	0.99	*CKM*	Thr166Met	4.3×10^{-9}	-0.165	6.00×10^{-9}	-0.165	0.02	-0.553
rs11559024	19	50,513,023	C/T	2.15	*CKM*	Glu83Gly	1.8×10^{-115}	-0.446	1.66×10^{-117}	-0.45	2.77×10^{-7}	-1.036
rs393600	19	59,450,914	G/A	25.17	*LILRB5*	-	1.4×10^{-33}	0.079	2.67×10^{-29}	0.091	2.85×10^{-18}	0.14
rs12975366	19	59,451,173	C/T	41.62	*LILRB5*	Asp147Gly, Asp247Gly	6.5×10^{-44}	-0.08	2.91×10^{-33}	-0.101	6.40×10^{-32}	-0.121

Supplementary Table 3: *Associations between LDH and all reported variants when testing alternative inheritance models*

Variant	Chr	Position (hg18)	Allele (min/maj)	MAF (%)	Gene[††]	Coding Change	Additive		Dominant		Recessive	
							P-value	Effect [Amin]	*P*-value	Effect [Amin]	*P*-value	Effect [Amin]
rs333947	1	110,272,287	A/G	14.92	*CSF1*	-	2.8×10^{-10}	-0.042	1.21×10^{-10}	-0.048	0.01	-0.058
rs2274700	1	194,949,570	A/G	38.62	*CFH*	-	4.1×10^{-12}	-0.034	2.47×10^{-10}	-0.043	1.11×10^{-7}	-0.048
rs150956780	3	52,526,606	C/G	0.078	*STAB1*	Val1522Leu	1.3×10^{-61}	-1.526	8.13×10^{-56}	-2.239	na	na
rs17412833	6	32,740,576	T/A	13.76	*HLA-DQB1*	Phe119Tyr	1.5×10^{-22}	0.07	4.00×10^{-22}	0.077	2.80×10^{-7}	0.127
rs12342201	9	94,934,785	A/G	49.45	*NINJ1*	-	1.1×10^{-12}	-0.034	1.26×10^{-11}	-0.052	9.54×10^{-8}	-0.04
rs61751507	10	101,819,504	T/C	4.06	*CPN1*	Gly178Asp	0.34	0.011	0.37	0.011	0.41	0.062
rs116841148	11	18,380,983	T/G	0.65	*LDHA*	Ala176Ser, Ala147Ser	2.9×10^{-11}	-0.198	1.05×10^{-11}	-0.2	na	na
rs7481951	11	22,228,446	A/T	37.45	*ANO5*	Leu322Phe	2.5×10^{-3}	-0.015	1.10×10^{-3}	-0.022	0.12	-0.015
rs137854526	11	22,240,353	C/T	0.24	*ANO5*	Phe578Ser	0.25	0.058	0.21	0.062	0.29	-0.828
chr11:22241070:S	11	22,241,070	A/T	0.27	*ANO5*	Cys601X	0.05	0.09	0.06	0.088	na	na
rs2930191	11	47,903,412	A/G	37.22	-	-	4.7×10^{-13}	-0.035	3.52×10^{-10}	-0.043	1.99×10^{-9}	-0.056
chr12:7282745:0:TA	12	7,282,745	TA/!TA	21.13	-	-	1.2×10^{-25}	-0.064	1.53×10^{-23}	-0.072	2.47×10^{-12}	-0.119
rs145411783	12	7,440,236	A/C	0.66	*CD163L1*	Arg601Leu	8.7×10^{-12}	0.203	4.98×10^{-12}	0.205	0.14	0.523
rs4072797	12	7,440,276	T/C	4.21	*CD163L1*	Asp588Asn	9.9×10^{-89}	-0.236	7.88×10^{-92}	-0.244	4.19×10^{-6}	-0.355
rs117692263	12	7,516,281	C/T	9.31	*CD163*	-	6.1×10^{-28}	0.09	9.39×10^{-27}	0.093	1.21×10^{-8}	0.202
rs4883263	12	7,540,751	T/C	3.73	*CD163*	Ile342Val	1.8×10^{-19}	0.114	1.20×10^{-19}	0.116	4.53×10^{-3}	0.266
rs7305678	12	7,572,448	T/G	16.16	-	-	2.0×10^{-18}	0.057	5.07×10^{-18}	0.063	4.27×10^{-6}	0.093
chr12:110830276:S	12	110,830,276	C/T	2.09	-	-	2.1×10^{-12}	-0.119	8.78×10^{-13}	-0.121	0.24	-0.191
rs7318906	13	73,004,132	A/G	47.08	-	-	2.9×10^{-3}	-0.014	0.05	-0.015	1.46×10^{-3}	-0.025
rs149354459	19	50,503,532	G/C	0.21	*CKM*	Arg251Pro	0.54	0.031	0.52	0.033	na	na
rs145987658	19	50,506,961	A/G	0.086	*CKM*	Thr180Met	0.58	0.047	0.74	0.023	0.72	-0.025
rs17357122	19	50,507,003	A/G	0.99	*CKM*	Thr166Met	0.45	0.018	0.46	0.018	0.74	0.069
rs11559024	19	50,513,023	C/T	2.15	*CKM*	Glu83Gly	0.16	0.023	0.15	0.024	0.85	0.036
rs393600	19	59,450,914	G/A	25.17	*LILRB5*	-	1.6×10^{-28}	0.062	4.51×10^{-25}	0.071	2.08×10^{-15}	0.11
rs12975366	19	59,451,173	C/T	41.62	*LILRB5*	Asp147Gly, Asp247Gly	7.0×10^{-51}	-0.074	5.28×10^{-42}	-0.098	1.34×10^{-32}	-0.105

Supplementary Table 4: *Pairwise r^2 and D' for sequence variants at loci harboring multiple signals. Shown are results for minor alleles, as per Table 1.*

Locus			MAF	MAF	r^2	Sign	D'
ANO5	rs7481951	chr11:22241070:S	37.5	0.27	0.0016	-1	1.00
	rs7481951	rs137854526	37.5	0.24	0.0014	-1	1.00
	chr11:22241070:S	rs137854526	0.27	0.24	$6x10^{-6}$	-1	1.00
CD163/ CD163L1	rs4072797	rs7305678	4.21	16.2	0.038	1	0.41
	rs4072797	chr12:7282745:0:TA	4.21	21.1	0.023	1	0.35
	rs4072797	rs145411783	4.21	0.66	0.00030	-1	1.00
	rs4072797	rs4883263	4.21	3.73	0.0017	-1	1.00
	rs4072797	rs117692263	4.21	9.31	0.0045	-1	1.00
	rs7305678	chr12:7282745:0:TA	16.2	21.1	$9.1x10^{-5}$	1	0.0093
	rs7305678	rs145411783	16.2	0.66	0.0013	-1	1.00
	rs7305678	rs4883263	16.2	3.73	0.030	1	0.39
	rs7305678	rs117692263	16.2	9.31	0.043	1	0.28
	chr12:7282745:0:TA	rs145411783	21.1	0.66	0.0019	-1	0.98
	chr12:7282745:0:TA	rs4883263	21.1	3.73	0.0078	-1	0.82
	chr12:7282745:0:TA	rs117692263	21.1	9.31	$2.5x10^{-5}$	1	0.0047
	rs145411783	rs4883263	0.66	3.73	0.016	1	0.32
	rs145411783	rs117692263	0.66	9.31	0.00069	-1	1.00
	rs4883263	rs117692263	3.73	9.31	0.00	-1	0.012
CKM	rs11559024	rs149354459	2.15	0.21	$4.7x10^{-5}$	-1	1.00
	rs11559024	rs17357122	2.15	0.99	0.00022	-1	1.00
	rs11559024	rs145987658	2.15	0.086	$2.2x10^{-5}$	-1	1.00
	rs149354459	rs17357122	0.21	0.99	$2.2x10^{-5}$	-1	1.00
	rs149354459	rs145987658	0.21	0.086	$6x10^{-6}$	-1	1.00
	rs17357122	rs145987658	0.99	0.086	$2.7x10^{-5}$	-1	1.00
LILRB5	rs12975366	rs393600	41.6	25.2	0.24	-1	1.00

Supplementary Table 5*: Variant frequencies (1000 Genomes Super Populations) and GERP Scores*

Variant	Position	Allele (min/maj)	MAF ICE (%)	MAF AFR (%)	MAF AMR (%)	MAF ASN (%)	MAF EUR (%)	GERP Score
rs333947	chr1:110272287	A/G	14.92	1.40	24.30	31.60	14.60	4.83
rs2274700	chr1:194949570	A/G	38.62	44.70	50.80	42.00	40.90	-1.53
rs150956780	chr3:52526606	C/G	0.078	-	-	-	0.40	4.56
rs17412833	chr6:32740576	T/A	13.76	29.90	27.90	21.00	33.10	-5.96
rs12342201	chr9:94934785	A/G	49.45	82.50	34.00	18.70	47.20	-2.69
rs61751507	chr10:101819504	T/C	4.06	0.20	6.10	2.80	4.10	4.16
rs116841148	chr11:18380983	T/G	0.65	-	-	-	0.40	5.30
rs7481951	chr11:22228446	A/T	37.45	89.60	58.00	79.50	41.70	2.87
chr11:22241070:S	chr11:22241070	A/T	0.27	-	-	-	-	4.41
rs137854526	chr11:22240353	C/T	0.24	-	-	-	0.10	5.69
rs2930191	chr11:47903412	A/G	37.22	21.60	48.10	29.70	38.60	0.18
rs4072797	chr12:7440276	T/C	4.21	29.70	9.70	17.00	3.20	-3.40
rs7305678	chr12:7572448	T/G	16.16	60.00	22.70	48.10	13.90	0
chr12:7282745:0:TA	chr12:7282745	TA/!TA	21.13	-	-	-	-	-0.093
rs145411783	chr12:7440236	A/C	0.66	-	-	-	0.70	-3.95
rs4883263	chr12:7540751	T/C	3.73	48.90	15.20	29.70	3.20	-3.37
rs117692263	chr12:7516281	C/T	9.31	3.90	6.10	10.10	9.10	0.19
chr12:110830276:S	chr12:110830276	C/T	2.09	-	-	-	-	0.14
rs7318906	chr13:73004132	A/G	47.08	27.60	57.20	26.40	48.50	-1.09
rs11559024	chr19:50513023	C/T	2.15	-	0.80	-	1.60	-8.54
rs149354459	chr19:50503532	G/C	0.21	-	-	-	0.10	5.04
rs17357122	chr19:50507003	A/G	0.99	0.20	0.60	-	0.80	4.08
rs145987658	chr19:50506961	A/G	0.17	-	-	-	-	4.61
rs12975366	chr19:59451173	C/T	41.62	18.50	37.30	7.70	41.20	1.69
rs393600	chr19:59450914	G/A	25.17	14.20	22.90	4.50	27.70	-2.84

*Variants not reported in 1000 Genomes

Population abbreviations: ICE = Inhouse data on Icelanders; AFR = African; AMR = Ad Mixed American; ASN = East Asian; EUR = European

Supplementary Table 6*: Single marker associations with serum aspartate transaminase levels*

Marker	Chr	Position	Gene	*P* value	Effect
rs333947	chr1	110,272,287	*CSF1*	**$8.2x10^{-10}$**	**-0.036**
rs2274700	chr1	194,949,570	*CFH*	0.02	-0.01
rs150956780	chr3	52,526,606	*STAB1*	0.68	0.052
rs17412833	chr6	32,740,576	*HLA-DQB1*	**$7.0x10^{-13}$**	**0.044**
rs12342201	chr9	94,934,785	*NINJ1*	0.11	-0.007
rs61751507	chr10	101,819,504	*CPN1*	0.59	-0.006
rs116841148	chr11	18,380,983	*LDHA*	0.80	-0.006
rs7481951	chr11	22,228,446	*ANO5*	**$4.7x10^{-4}$**	**-0.015**
rs137854526	chr11	22,240,353	*ANO5*	0.36	0.04
chr11:22241070:S	chr11	22,241,070	*ANO5*	0.05	0.078
rs2930191	chr11	47,903,412	-	**$8.8x10^{-5}$**	**-0.017**
chr12:7282745:0:TA	chr12	7,282,745	-	0.28	-0.006
rs145411783	chr12	7,440,236	*CD163L1*	0.88	0.004
rs4072797	chr12	7,440,276	*CD163L1*	0.95	-0.001
rs117692263	chr12	7,516,281	*CD163*	0.29	0.007
rs4883263	chr12	7,540,751	*CD163*	0.72	0.004
rs7305678	chr12	7,572,448	-	0.29	0.006
chr12:110830276:S	chr12	110,830,276	-	**$4.4x10^{-5}$**	**-0.059**
rs7318906	chr13	73,004,132	-	0.02	-0.01
rs149354459	chr19	50,503,532	*CKM*	0.10	0.074
rs145987658	chr19	50,506,961	*CKM*	0.30	0.067
rs17357122	chr19	50,507,003	*CKM*	0.36	-0.019
rs11559024	chr19	50,513,023	*CKM*	0.98	0
rs393600	chr19	59,450,914	*LILRB5*	**$4.2x10^{-4}$**	**0.017**
rs12975366	chr19	59,451,173	*LILRB5*	**$6.0x10^{-6}$**	**-0.019**

Supplementary Table 7: *Phenotypes observed in homozygous carriers of sequence variants in ANO5, obtained from Icelandic hopital discharge records*

	Homozygous for Sequence Variant	ICD10 Code	Phenotype
Case 1	rs137854526 (Phe578Ser)	I20	Angina pectoris
		I251	Athoerosclerotic heart disease
		I259	Chronic ischaemic heart disease
		I500	Congestive heart failure
Case 2	chr11:22241070 (Cys601X)	I259	Chronic ischaemic heart disease
		I509	Heart failure

Supplementary Table 8: *Heterogeneity of effects of identified sequence variants on serum lactate dehydrogenase (LDH) levels when statin users are assessed either during or outside of times of statin usage. Significance was determined by the number of tests performed (0.05/26 = $1.9x10^{-3}$). No significant heterogeneity was observed.*

	Effect on CK (All)		Effect on CK During Statin Use		Effect on CK Outside of Statin Use		Effect Heterogeneity (On Statin vs. Off Statin)	
**Marker**	***P***	**Effect**	***P***	**Effect**	***P***	**Effect**	**HetP**	**I^2**
rs333947	$6.8x10^{-3}$	-0.021	0.37	-0.02	0.94	0.001	0.42	0
rs2274700	0.58	-0.003	0.91	-0.002	0.13	0.02	0.31	3.3
rs150956780	0.60	0.05	0.5	-0.305	0.39	0.388	0.28	15
rs17412833	$1.5x10^{-4}$	0.032	0.14	0.035	0.42	0.016	0.54	0
rs12342201	0.70	0.002	0.51	-0.01	0.44	-0.01	1	0
rs61751507	$5.1x10^{-11}$	-0.091	0.44	-0.032	0.02	-0.078	0.39	0
rs116841148	0.17	0.049	0.07	0.2	0.11	0.134	0.64	0
rs7481951	$6.4x10^{-16}$	-0.047	0.0089	-0.043	0.0018	-0.043	1	0
rs137854526	$4.5x10^{-4}$	0.204	0.36	0.132	0.35	0.115	0.93	0
chr11:22241070:S	$3.9x10^{-6}$	0.245	0.26	0.151	0.061	0.199	0.78	0
rs2930191	$2.3x10^{-4}$	-0.021	0.84	0.003	0.067	-0.024	0.17	47
chr12:7282745:0:TA	0.49	-0.005	0.84	-0.004	0.081	-0.029	0.33	0
rs145411783	0.90	0.005	0.85	-0.019	0.66	-0.036	0.89	0
rs4072797	0.63	0.007	0.37	-0.035	0.29	-0.034	0.98	0
rs117692263	$1.2x10^{-17}$	0.083	0.04	0.055	0.097	0.037	0.6	0
rs4883263	$5.7x10^{-5}$	0.059	0.57	0.023	0.25	0.04	0.75	0
rs7305678	$3.0x10^{-21}$	0.072	0.00012	0.083	$8.1X10^{-5}$	0.07	0.64	0
chr12:110830276:S	$2.9x10^{-4}$	-0.071	0.083	-0.099	0.00013	-0.177	0.29	11.1
rs7318906	$2.7x10^{-13}$	-0.041	0.00082	-0.053	0.15	-0.019	0.1	63.1
rs149354459	$6.0x10^{-20}$	-0.55	0.38	-0.151	0.0011	-0.494	0.14	55.2
rs145987658	$8.9x10^{-9}$	-0.59	0.45	-0.211	0.16	-0.353	0.7	0
rs17357122	$4.3x10^{-9}$	-0.165	0.0019	-0.242	0.012	-0.158	0.4	0
rs11559024	$1.8x10^{-115}$	-0.446	$5.2X10^{-18}$	-0.486	$1.4X10^{-23}$	-0.468	0.81	0
rs393600	$1.4x10^{-33}$	0.079	$8.6X10^{-10}$	0.113	$8.4X10^{-11}$	0.099	0.56	0
rs12975366	$6.5x10^{-44}$	-0.08	$2.1X10^{-10}$	-0.104	$9.2X10^{-15}$	-0.104	1	0

Supplementary Table 9: *Heterogeneity of effects of identified sequence variants on serum lactate dehydrogenase (LDH) levels when statin users are assessed either during or outside of times of statin usage. Significance was determined by the number of tests performed (0.05/26 = 1.9x10^{-3}). No significant heterogeneity was observed.*

	Effect on LDH (All)		Effect on LDH During Statin Use		Effect on LDH Outside of Statin Use		Effect Heterogeneity (On Statin vs. Off Statin)	
**Marker**	***P***	**Effect**	***P***	**Effect**	***P***	**Effect**	**HetP**	**I^2**
rs333947	2.8x10^{-10}	-0.042	5.6x10^{-4}	-0.071	8.7x10^{-4}	-0.060	0.69	0
rs2274700	4.1x10^{-12}	-0.034	0.0030	-0.045	0.085	-0.023	0.28	15.7
rs150956780	1.3x10^{-61}	-1.526	1.1x10^{-5}	-2.304	1.3x10^{-11}	-2.6	0.65	0
rs17412833	1.5x10^{-22}	0.070	0.0028	0.064	0.025	0.043	0.47	0
rs12342201	1.1x10^{-12}	-0.034	0.68	-0.006	0.0048	-0.036	0.12	58.9
rs61751507	0.34	0.011	0.88	-0.006	0.35	0.03	0.49	0
rs116841148	2.9x10^{-11}	-0.198	0.045	-0.204	0.090	-0.144	0.65	0
rs7481951	2.5x10^{-3}	-0.015	0.18	-0.021	0.065	-0.025	0.85	0
rs137854526	0.25	0.058	0.13	-0.213	0.43	-0.10	0.55	0
chr11:22241070:S	0.05	0.09	0.96	0.006	0.14	0.16	0.35	0
rs2930191	4.7x10^{-13}	-0.035	0.091	-0.025	0.0037	-0.038	0.51	0
chr12:7282745:0:TA	1.2x10^{-25}	-0.064	2.4x10^{-4}	-0.069	1.3x10^{-6}	-0.08	0.66	0
rs145411783	8.7x10^{-12}	0.203	0.43	0.069	0.0099	0.197	0.27	17.8
rs4072797	9.9x10^{-89}	-0.236	7.0x10^{-9}	-0.206	2.2x10^{-18}	-0.276	0.14	53.9
rs117692263	6.1x10^{-28}	0.090	0.0041	0.072	0.022	0.051	0.53	0
rs4883263	1.8x10^{-19}	0.114	0.026	0.086	0.0026	0.102	0.76	0
rs7305678	2.0x10^{-18}	0.057	0.0076	0.053	0.042	0.036	0.52	0
chr12:110830276:S	2.1x10^{-12}	-0.119	0.0015	-0.17	0.0016	-0.148	0.76	0
rs7318906	2.9x10^{-3}	-0.014	0.45	-0.011	0.93	0.001	0.51	0
rs149354459	0.54	0.031	0.47	0.104	0.40	0.116	0.95	0
rs145987658	0.58	0.047	0.66	0.102	0.94	-0.019	0.72	0
rs17357122	0.45	0.018	0.22	-0.091	0.24	-0.071	0.84	0
rs11559024	0.16	0.023	0.99	-0.001	0.098	0.076	0.29	9.9
rs393600	1.6x10^{-28}	0.062	1.7x10^{-6}	0.082	7.6x10^{-7}	0.075	0.76	0
rs12975366	7.0x10^{-51}	-0.074	1.5x10^{-5}	-0.066	2.1x10^{-8}	-0.075	0.66	0

Supplementary Table 10: *Associations between known variant rs4363657 in SLCO1B1 and all tested phenotypes*

Pval	Effect	Phenotype
0.26	0.008	Creatine Kinase
0.35	0.019	Creatine Kinase (On Statin)
0.06	0.033	Creatine Kinase (Off Statin)
0.91	0.001	Lactate Dehydrogenase
0.45	-0.015	Lactate Dehydrogenase (On Statin)
0.87	-0.003	Lactate Dehydrogenase (Off Statin)

Supplementary Table 11: *Correlations between genotypes of WGS Icelanders and their imputed genotypes using the 1000 Genomes imputation set (Phase I) for 20 markers present in both sets reported in this manuscript*

Marker	Position	Alleles [min/maj]	r^2 (Imputed vs. Real)
rs333947	chr1:110272287	[G/A]	0.91
rs2274700	chr1:194949570	[G/A]	0.99
rs150956780	chr3:52526606	[G/C]	0
rs17412833	chr6:32740576	[A/T]	NA*
rs12342201	chr9:94934785	[G/A]	0.96
rs61751507	chr10:101819504	[C/T]	0.94
rs116841148	chr11:18380983	[G/T]	0.51
rs137854526	chr11:22240353	[T/C]	NA*
rs2930191	chr11:47903412	[G/A]	0.95
rs145411783	chr12:7440236	[C/A]	0.97
rs4072797	chr12:7440276	[C/T]	0.99
rs117692263	chr12:7516281	[T/C]	0.96
rs4883263	chr12:7540751	[T/C]	0.83
rs7305678	chr12:7572448	[T/G]	0.99
rs7318906	chr13:73004132	[G/A]	1
rs149354459	chr19:50503532	[C/G]	NA*
rs17357122	chr19:50507003	[G/A]	0.8
rs11559024	chr19:50513023	[T/C]	0.95
rs393600	chr19:59450914	[G/A]	0.85
rs12975366	chr19:59451173	[T/C]	0.63

** NA = markers not present in 1000G Phase I but present in 1000G Phase 3 data.*

Supplementary Table 12: *Correlates of reported markers, based on Icelandic data, that are themselves not present in the 1000 genomes reference panel, and the degrees of correlation between their directly typed and imputed genotypes in the 1000 Genomes reference panel. For five markers reported in this manuscript.*

Reported Variant	Correlate	Alleles [min/maj]	MAF (%)	r^2	D'	r^2 (Imputed vs. Real)
chr11:22228446:S	chr11:22132253:S	[G/T]	44.6	0.63	1	0.83
	chr11:22132254:S	[A/T]	44.6	0.63	1	0.83
	chr11:22132480:I	[C/CCAAA]	44.6	0.63	1	NA
	chr11:22134340:S	[C/T]	43.5	0.67	1	0.89
	chr11:22135253:S	[T/C]	38.9	0.82	1	0.98
	chr11:22135551:S	[A/G]	38.9	0.82	1	0.98
	chr11:22136569:S	[T/C]	38.9	0.82	1	0.98
	chr11:22136883:S	[A/G]	38.9	0.82	1	0.98
	chr11:22138940:S	[T/C]	38.9	0.82	1	0.99
	chr11:22155841:S	[A/G]	40.2	0.82	1	0.94
	chr11:22157339:S	[G/A]	43.9	0.70	1	0.96
	chr11:22157583:S	[T/G]	39.6	0.84	1	0.96
	chr11:22177816:S	[C/A]	41.4	0.80	1	0.98
	chr11:22183424:S	[A/G]	41.0	0.81	1	0.92
	chr11:22183460:S	[T/C]	41.0	0.81	1	0.92
	chr11:22212062:S	[G/A]	27.1	0.62	1	0.93
	chr11:22239363:S	[G/A]	48.4	0.61	1	0.86
	chr11:22246097:S	[G/A]	48.9	0.63		0.87
chr12:7282745:0:TA	chr12:7267513:S	[C/T]	20.7	0.72	1	1
	chr12:7274848:S	[T/C]	20.7	0.73	1	NA
	chr12:7286581:S	[A/G]	22.0	0.68	1	1
	chr12:7288524:S	[G/A]	21.7	0.67	1	0.99
	chr12:7300178:S	[G/T]	21.7	0.67	1	0.72
	chr12:7301165:S	[A/C]	21.8	0.67	1	0.99
chr12:110830276:S	chr12:110373191:S	[C/T]	2.8	0.75	-1	0.9
	chr12:110385162:S	[C/T]	3.2	0.79	-1	NA
	chr12:110441793:S	[T/C]	2.8	0.76	-1	0.87
	chr12:110462920:S	[T/C]	2.3	0.90	-1	NA
	chr12:110555332:S	[A/G]	2.7	0.76	-1	0.7
	chr12:110566902:S	[T/C]	2.8	0.76	-1	0.87
	chr12:110648651:S	[G/A]	2.8	0.75	-1	0.9
	chr12:111045555:S	[A/G]	2.6	0.80	-1	0.89
	chr12:111057324:S	[A/G]	2.8	0.75	-1	0.89
	chr12:111117509:S	[T/C]	2.7	0.76	-1	0.87
	chr12:111197154:S	[G/A]	2.8	0.75	-1	0.89
	chr12:111290544:S	[A/G]	2.1	1.00	-1	0

	chr12:111301047:S	[A/G]	2.1	1.00	-1	NA
chr19:50506961:S	chr19:50714764:S	[A/G]	0.1	0.70	-1	0
chr19:59451173:S	chr19:59444029:S	[G/C]	41.2	0.66	-1	0.72
	chr19:59445014:S	[C/T]	41.3	0.67	-1	0.71
	chr19:59451478:S	[C/T]	44.2	0.71	-1	0.66

Supplementary Table 13: *Characteristics of CK and LDH measurements among the Icelandic laboratories.*

	RAM	FSA	LUH
Number of CK Measurements	25,152	7,867	219,569
Unique Individuals	14,961	3,966	49,467
% Females	59.8	48.3	47.5
Median CK (Quantiles: 25%, 75%)	96.0 (68.0, 142.0)	98.0 (65.0, 155.0)	90.0 (49.0, 219.0)
Geometric Mean	101.0	109.4	114.7
Number of LDH Measurements	24,378	20,103	445,744
Unique Individuals	4,677	7,346	95,372
% Females	47.4	55.0	54.4
Median LDH (Quantiles: 25%, 75%)	190.0 (169.0, 215.0)	185.0 (160.0, 220.0)	195.0 (164.5, 244.0)
Geometric Mean	192.6	196.0	212.9

RAM: The Icelandic Medical Center (Laeknasetrid) Laboratory in Mjodd.
LUH: Landspitali - The National University Hospital of Iceland.
FSA: Akureyri Hospital

Supplementary Note 1

Statistical analysis

Long range phasing

Long range phasing of all chip-genotyped individuals was performed with methods described previously[1-5]. In brief, phasing is achieved using an iterative algorithm which phases a single proband at a time given the available phasing information about everyone else that shares a long haplotype identically by state with the proband. Given the large fraction of the Icelandic population that has been chip-typed, accurate long range phasing is available genome-wide for all chip-typed Icelanders.

Genotype imputation

We imputed the SNPs identified and genotyped through sequencing into all Icelanders who had been phased with long range phasing using the same model as used by IMPUTE[6]. The genotype data from sequencing can be ambiguous due to low sequencing coverage. In order to phase the sequencing genotypes, an iterative algorithm was applied for each SNP with alleles 0 and 1. We let H be the long range phased haplotypes of the sequenced individuals and applied the following algorithm:

1. For each haplotype h in H, use the Hidden Markov Model of IMPUTE to calculate for every other k in H, the likelihood, denoted $\gamma_{h,k}$, of h having the same ancestral source as k at the SNP. For every h in H, initialize the parameter ϑ_h, which specifies how likely the one allele of the SNP is to occur on the background of h from the genotype likelihoods obtained from sequencing. The genotype likelihood L_g is the probability of the observed sequencing data at the SNP for a given individual assuming g is the true genotype at the SNP. If L_0, L_1 and L_2 are the likelihoods of the genotypes 0, 1 and 2 in the individual that carries h, then set

$\theta_h = \frac{L_2+\frac{1}{2}L_1}{L_2+L_1+L_0}$.

2. For every pair of haplotypes *h* and *k* in *H* that are carried by the same individual, use the other haplotypes in *H* to predict the genotype of the SNP on the backgrounds of *h* and *k*: $\tau_h = \sum_{l\in H\setminus\{h\}} \gamma_{h,l}\theta_l$ and $\tau_k = \sum_{l\in H\setminus\{k\}} \gamma_{k,l}\theta_l$. Combining these predictions with the genotype likelihoods from sequencing gives un-normalized updated phased genotype probabilities: $P_{00} = (1-\tau_h)(1-\tau_k)L_0$, $P_{10} = \tau_h(1-\tau_k)\frac{1}{2}L_1$, $P_{01} = (1-\tau_h)\tau_k\frac{1}{2}L_1$ and $P_{11} = \tau_h\tau_k L_2$.
3. Now use these values to update ϑ_h and ϑ_k to $\theta_h = \frac{P_{10}+P_{11}}{P_{00}+P_{01}+P_{10}+P_{11}}$ and $\theta_k = \frac{P_{01}+P_{11}}{P_{00}+P_{01}+P_{10}+P_{11}}$.
4. Repeat step 3 when the maximum difference between iterations is greater than a convergence threshold ε. We used $\varepsilon=10^{-7}$.

Given the long range phased haplotypes and ϑ, the allele of the SNP on a new haplotype *h* not in *H*, is imputed as $\sum_{l\in H} \gamma_{h,l}\theta_l$.

The above algorithm can easily be extended to handle simple family structures such as parent-offspring pairs and triads by letting the *P* distribution run over all founder haplotypes in the family structure. The algorithm also extends trivially to the X-chromosome. If source genotype data are only ambiguous in phase, such as chip genotype data, then the algorithm is still applied, but all but one of the *L*s will be 0. In some instances, the reference set was intentionally enriched for carriers of the minor allele of a rare SNP in order to improve imputation accuracy. In this case, expected allele counts will be biased toward the minor allele of the SNP. Call the enrichment of the minor allele *E* and let ϑ' be the expected minor allele count calculated from the naïve imputation method, and let ϑ be the unbiased expected allele count, then $\theta' = \frac{E\theta}{1-\theta+E\theta}$ and hence $\theta = \frac{\theta'}{E+(1-E)\theta'}$.

This adjustment was applied to all imputations based on enriched imputations sets. We note that if ϑ' is 0 or 1, then ϑ will also be 0 or 1, respectively.

Genotype Imputation of Unchipped Relatives

In addition to imputing sequence variants from the whole genome sequencing effort into chip genotyped individuals, we also performed a second imputation step where genotypes were imputed into relatives of chip genotyped individuals. These genotypes were created on the fly and were not stored. The inputs into the second imputation step are the fully phased (in particular every allele has been assigned a parent of origin) imputed and chip type genotypes of the available chip typed individuals. The algorithm used to perform the second imputation step consists of:

1. For each ungenotyped individual (the proband), find all chip genotyped individuals within two meiosis of the individual. The six possible types of two meiosis relatives of the proband are (ignoring more complicated relationships due to pedigree loops): Parents, full and half siblings, grandparents, children and grandchildren. If all pedigree paths from the proband to a genotyped relative go through other genotyped relatives, then that relative is excluded. E.g. if a parent of the proband is genotyped, then the proband's grandparents through that parent are excluded. If the number of meiosis in the pedigree around the proband exceeds a threshold (we used 12), then relatives are removed from the pedigree until the number of meiosis falls below 12, in order to reduce computational complexity.
2. At every point in the genome, calculate the probability for each genotyped relative sharing with the proband based on the autosomal SNPs used for phasing. A multipoint algorithm based on the hidden Markov model Lander-Green multipoint linkage algorithm using fast Fourier transforms is used to calculate these sharing probabilities. First single point sharing probabilities are calculated by dividing the genome into 0.5cM bins and using the haplotypes over these bins as alleles. Haplotypes that are the same, except at most at a single SNP, are

treated as identical. When the haplotypes in the pedigree are incompatible over a bin, then a uniform probability distribution was used for that bin. The most common causes for such incompatibilities are recombinations in member belonging to the pedigree, phasing errors and genotyping errors. Note that since the input genotypes are fully phased, the single point information is substantially more informative than for unphased genotyped, in particular one haplotype of the parent of a genotyped child is always known. The single point distributions are then convolved using the multipoint algorithm to obtain multipoint sharing probabilities at the center of each bin. Genetic distances were obtained from the most recent version of the deCODE genetic map[2].

3. Based on the sharing probabilities at the center of each bin, all the SNPs from the whole genome sequencing are imputed into the proband. To impute the genotype of the paternal allele of a SNP located at x, flanked by bins with centers at x_{left} and x_{right}. Starting with the left bin, going through all possible sharing patterns v, let I_v be the set of haplotypes of genotyped individuals that share identically by descent within the pedigree with the proband's paternal haplotype given the sharing pattern v and $P(v)$ be the probability of v at the left bin – this is the output from step 2 above – and let e_i be the expected allele count of the SNP for haplotype i. Then $e_v = \frac{\sum_{i \in I_v} e_i}{\sum_{i \in I_v} 1}$ is the expected allele count of the paternal haplotype of the proband given v and an overall estimate of the allele count given the sharing distribution at the left bin is obtained from $e_{left} = \sum_v P(v) e_v$. If I_v is empty then no relative shares with the proband's paternal haplotype given v and thus there is no information about the allele count. We therefore store the probability that some genotyped relative shared the proband's paternal haplotype, $O_{left} = \sum_{v, I_v = \emptyset} P(V)$ and an expected allele count, conditional on the proband's paternal haplotype being shared by at least one genotyped relative: $c_{left} = \frac{\sum_{v, I_v \neq \emptyset} P(v) e_v}{\sum_{v, I_v \neq \emptyset} P(v)}$. In the same way calculate O_{right} and c_{right}. Linear interpolation is then used to get an estimates at the SNP from the two flanking bins:

$$O = O_{left} + \frac{x - x_{left}}{x_{right} - x_{left}}(O_{right} - O_{left}),$$

$$c = c_{left} + \frac{x - x_{left}}{x_{right} - x_{left}}(c_{right} - c_{left}).$$

If θ is an estimate of the population frequency of the SNP then $Oc + (1 - O)\theta$ is an estimate of the allele count for the proband's paternal haplotype. Similarly, an expected allele count can be obtained for the proband's maternal haplotype.

Genotype imputation information. The informativeness of genotype imputation was estimated by the ratio of the variance of imputed expected allele counts and the variance of the actual allele counts:

$$\frac{Var(E(\theta|chip\ data))}{Var(\theta)},$$

where $\theta \in \{0, 1\}$ is the allele count. $Var(E(\theta|chip\ data))$ was estimated by the observed variance of the imputed expected counts and $Var(\theta)$ was estimated by $p(1 - p)$, where p is the allele frequency. For the present study, when imputed genotypes are used, the information value for all SNPs is between 0.92 and 0.99.

Quantitative trait association testing

A generalized form of linear regression was used to test for association of CK and LDH levels with SNPs. Let y be the vector of quantitative measurements, and let g be the vector of expected allele counts for the SNP being tested. We assume the quantitative measurements follow a normal distribution with a mean that depends linearly on the expected allele at the SNP and a variance covariance matrix proportional to the kinship matrix:

$$y \sim \mathcal{N}(\alpha + \beta g, 2\sigma^2\Phi),$$

where

$$\Phi_{ij} = \begin{cases} \frac{1}{2}, & i = j \\ 2k_{ij}, & i \neq j \end{cases}.$$

is the kinship matrix as estimated from the Icelandic genealogical database. It is not computationally feasible to use this full model and we therefore split the individuals with in silico genotypes and CK/LDH measurements into smaller clusters. Here we chose to restrict the cluster size to at most 300 individuals.

The maximum likelihood estimates for the parameters α, β, and σ^2 involve inverting the kinship matrix. If there are n individuals in the cluster, then this inversion requires $O(n^3)$ calculations, but since these calculations only need to be performed once the computational cost of doing a genome-wide association scan will only be $O(n^2)$ calculations; the cost of calculating the maximum likelihood estimates if the kinship matrix has already been inverted.

Supplementary References

1. Kong, A. et al. Detection of sharing by descent, long-range phasing and haplotype imputation. *Nat Genet* **40**, 1068-75 (2008).
2. Kong, A. et al. Fine-scale recombination rate differences between sexes, populations and individuals. *Nature* **467**, 1099-103 (2010).
3. Sulem, P. et al. Identification of low-frequency variants associated with gout and serum uric acid levels. *Nat Genet* **43**, 1127-30 (2011).
4. Rafnar, T. et al. Mutations in BRIP1 confer high risk of ovarian cancer. *Nat Genet* **43**, 1104-7 (2011).
5. Stacey, S.N. et al. A germline variant in the TP53 polyadenylation signal confers cancer susceptibility. *Nat Genet* **43**, 1098-103 (2011).
6. Marchini, J., Howie, B., Myers, S., McVean, G. & Donnelly, P. A new multipoint method for genome-wide association studies by imputation of genotypes. *Nat Genet* **39**, 906-13 (2007).
